# Targeting NSD2-mediated SRC-3 liquid–liquid phase separation sensitizes bortezomib treatment in multiple myeloma

**DOI:** 10.1038/s41467-021-21386-y

**Published:** 2021-02-15

**Authors:** Jing Liu, Ying Xie, Jing Guo, Xin Li, Jingjing Wang, Hongmei Jiang, Ziyi Peng, Jingya Wang, Sheng Wang, Qian Li, Linquan Ye, Yuping Zhong, Qiguo Zhang, Xiaozhi Liu, David M. Lonard, Jin Wang, Bert W. O’Malley, Zhiqiang Liu

**Affiliations:** 1 The province and ministry co-sponsored collaborative innovation center for medical epigenetics; Tianjin Key Laboratory of Cellular Homeostasis and Human Diseases; Department of Physiology and Pathophysiology, School of Basic Medical Science, Tianjin Medical University, Heping Tianjin, China; 2 Tianjin Medical University Cancer Institute and Hospital; National Clinical Research Center for Cancer; Tianjin Key Laboratory of Cancer Prevention and Therapy; Tianjin’s Clinical Research Center for Cancer, Tianjin, China; 3 Center for Translational Research in Hematological Malignancies, Cancer Center, Houston Methodist Hospital, Houston, TX USA; 4 Department of Hematology, Myeloma Research Center of Beijing, Beijing Chao-Yang Hospital, Capital Medical University, Chaoyang Beijing, China; 5 Department of Hematology, the Affiliated Nanjing Drum Tower Hospital of Nanjing University Medical School, Nanjing, China; 6 Central Laboratory, Tianjin Key Laboratory of Epigenetics for Organ Development of Premature Infants, The Fifth Central Hospital of Tianjin, Tianjin, China; 7 Department of Molecular and Cellular Biology, Baylor College of Medicine, Baylor College of Medicine, Houston, TX USA; 8 Department of Pharmacology and Chemical Biology, Houston, TX USA

**Keywords:** Haematological cancer, Myeloma, Chromatin, Chromatin remodelling, Epigenetics

## Abstract

Development of chemoresistance is the main reason for failure of clinical management of multiple myeloma (MM), but the genetic and epigenetic aberrations that interact to confer such chemoresistance remains unknown. In the present study, we find that high steroid receptor coactivator-3 (SRC-3) expression is correlated with relapse/refractory and poor outcomes in MM patients treated with bortezomib (BTZ)-based regimens. Furthermore, in immortalized cell lines, high SRC-3 enhances resistance to proteasome inhibitor (PI)-induced apoptosis. Overexpressed histone methyltransferase NSD2 in patients bearing a t(4;14) translocation or in BTZ-resistant MM cells coordinates elevated SRC-3 by enhancing its liquid–liquid phase separation to supranormally modify histone H3 lysine 36 dimethylation (H3K36me2) modifications on promoters of anti-apoptotic genes. Targeting SRC-3 or interference of its interactions with NSD2 using a newly developed inhibitor, SI-2, sensitizes BTZ treatment and overcomes drug resistance both in vitro and in vivo. Taken together, our findings elucidate a previously unrecognized orchestration of SRC-3 and NSD2 in acquired drug resistance of MM and suggest that SI-2 may be efficacious for overcoming drug resistance in MM patients.

## Abstract (web summary)

The mechanisms behind acquired resistance to the proteasome inhibitor bortezomib in multiple myeloma remain to be elucidated. Here, the authors show that the histone methyltransferase NSD2 stabilized SRC-3 protein levels, promotes its phase separation and alters H3K36me2 at certain gene promoters resulting in a transcriptional profile that favors resistance of myeloma cells to bortezomib.

## Introduction

Multiple myeloma (MM) is the second-leading adult hematologic malignancy and is characterized by uncontrolled expansion of malignant plasma cells primarily in bone marrow^1^, accounting for approximately 15% of hematological malignancies and 1.3% of the total cancer occurrence worldwide^2^. As the aging population has increased over time, the incidence of MM has increased to approximately 86,000 annual cases globally, leading to considerable clinical and economic burdens^3^. Despite considerable progress in the development of novel anti-myeloma agents and treatment strategies—including proteasome inhibitors, immunomodulatory agents, histone deacetylase inhibitors^4^, monoclonal antibodies, cellular therapy (CAR-T), and stem cell transplantation in fit patients^5,6^—nearly all patients eventually relapse and often develop multidrug resistance. Therefore, it is critical to further elucidate the mechanisms underlying acquired drug resistance to clinically available drugs in order to develop more effective therapies.

Genetic abnormalities and epigenetic aberrations play crucial roles in MM pathogenesis. Chromosomal abnormalities—such as copy-number variations, translocations, and mutations—are crucial primary events for MM initiation^7^. Additionally, aberrant epigenetic landscapes such as DNA methylation and histone modifications further contribute to MM disease progression, clonal heterogeneity, cellular plasticity, and drug resistance^8^. Importantly, genetic and epigenetic aberrations have been shown to orchestrally regulate genomic instability and transcriptomic alterations, which contribute to dedifferentiation of malignant cells to a less mature status that can lead to drug resistance and poor disease progression^8,9^. It has been well elucidated that abnormal histone methylation also plays an important role in MM pathogenesis, as high levels of histone methyltransferases (HMTs) and demethylases (HDMs) have been found in patients with genetic mutations^10^. For example, chromosomal translocation t(4;14)(p16;q32) is detected in 10–15% of MM patients and leads to overexpression of fibroblast growth factor receptor 3 (*FGFR3*) and multiple myeloma SET domain (*MMSET*)^11^. *MMSET* encodes a histone methyltransferase NSD2 that mainly catalyzes histone H3 lysine 36 dimethylation (H3K36me2) to consequently mediate chromatin structure remodeling, activation of gene transcription, DNA repair, and cellular survival^12,13^. Moreover, overexpression of NSD2 has been identified as an adverse prognostic marker for MM, and recent studies have revealed that NSD2 enhances drug resistance of MM through H4K20me3-mediated recruitment of 53BP1 and enhanced repair of DNA damage^14^. As a consequence, the accumulation of H3K36me2 triggers an altered transcriptome that favors cellular survival through redistribution of methyltransferase KMT6A/EZH2^15^. In addition, NSD2 enhances anti-apoptotic effects in diverse cancers by modulating non-histone oncoproteins, such as p53 and Aurora A protein, consequentially regulating anti-apoptotic factors, such as MCL1 and BCL2^16,17^. Despite these substantial findings on the genetics of MM, the mechanisms of NSD2-mediated acquisition of drug resistance remain unknown.

Steroid receptor coactivator-3 (SRC-3) belongs to the SRC/p160 coactivator family, which consists of three members: SRC-1/NCOA1, SRC-2/TIF2/GRIP1/NCOA2, and SRC-3/AIB1/NCOA3. SRC-3 is amplified and overexpressed in diverse human cancers. Moreover, clinical studies and cellular experiments have demonstrated that SRC-3 promotes numerous aspects of cancer—such as cancer initiation, progression, and chemoresistance—through the integration of nuclear hormone receptors (or other transcription factors) and multiple cancer-growth pathways^18^. Recent evidence has revealed that amplification of the *NCOA3* gene is highly associated with primary chemoresistance in ovarian cancers, suggesting that SRC-3 is most likely a driver in chemoresistance^19^. Additionally, overexpression of SRC-3 is associated with tamoxifen resistance and leads to markedly negative clinical outcomes in breast cancer^20^, to platinum resistance of ovarian epithelial cancer^21^, and resistance to cytotoxic agents in breast cancer^22^. Consistent with this interpretation, suppression of SRC-3 protein levels has been shown to sensitize anticancer reagents to effectively treat prostate cancer and leukemia cells^23,24^. Taken together, these findings have elucidated the importance of SRC-3 in primary and acquired drug resistance in various cancers. Nevertheless, it remains unclear whether and how SRC-3 is involved in drug resistance in MM, especially in terms of its integration with epigenetic regulators, such as NSD2. Achieving a better understanding of the roles and mechanisms of how SRC-3 is involved in MM drug resistance, as well as corresponding developments of novel therapeutic strategies, may help to better treat MM in the future.

In the current study, we investigated the roles of SRC-3 in response to treatment with proteasome inhibitors in myeloma cells. Furthermore, we explored the underlying mechanisms of SRC-3-mediated chromatin remodeling and transcriptomic alterations, and evaluated the efficacy of a newly developed SRC-3 inhibitor, SI-2, in overcoming bortezomib (BTZ) resistance in myeloma cells both in vitro and in vivo.

## Results

### SRC-3 is associated with clinical treatment outcomes and bone-lesion restoration in MM patients

**Fig. 1:**
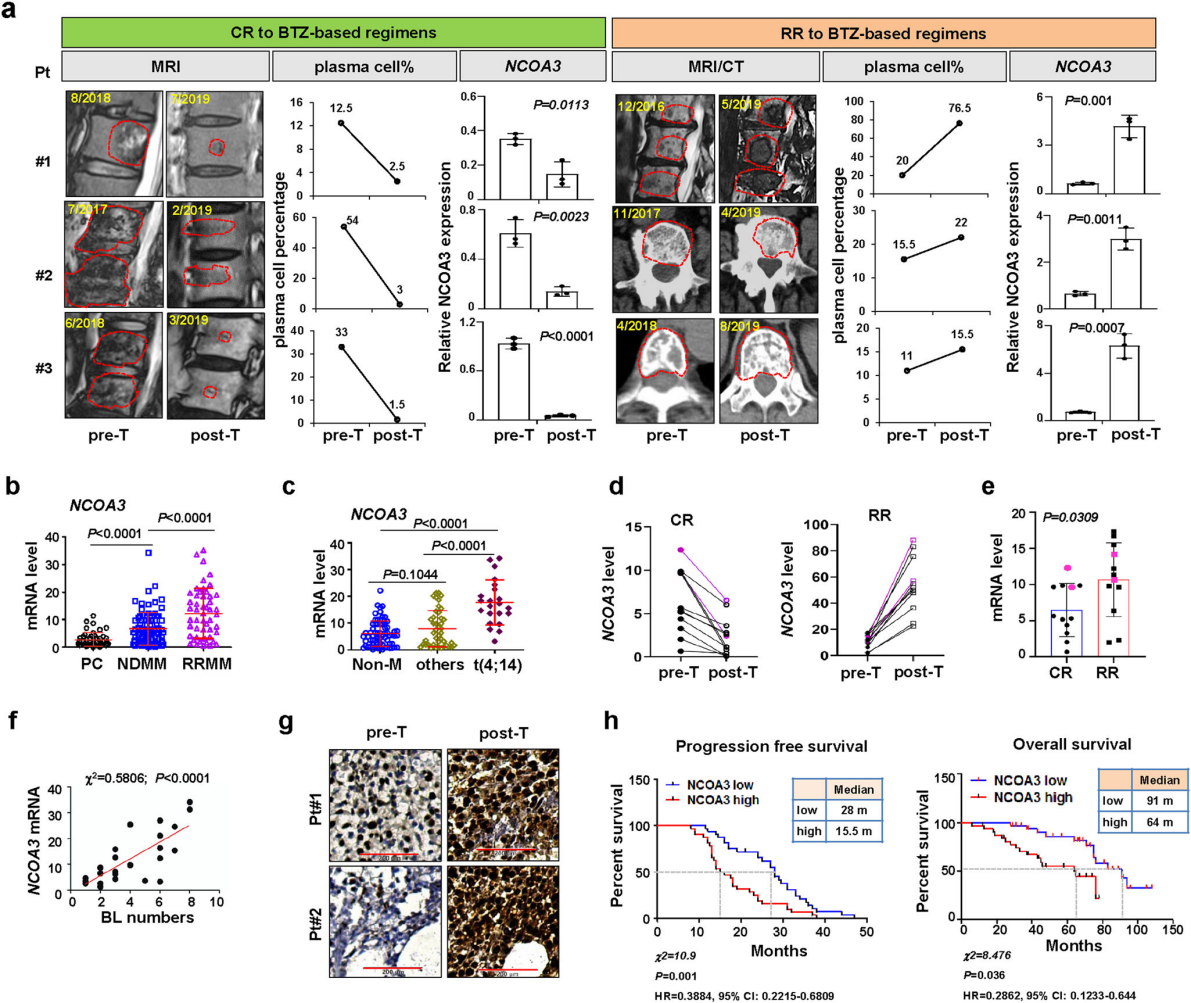
SRC-3 level correlates with treatment outcomes of MM patients in clinic. **a** Histological views of bone disruption, percentage of plasma cells (*n* = 3 biological independent samples), and SRC-3 (*NCOA3* as gene nomenclature) expressions (mean ± SD, *n* = 3 biologically independent experiments) in bone marrow biopsies from patients with CR or RR before (pre-T) and after (post-T) bortezomib (BTZ) based treatments. MRI, magnetic resonance imaging; CT, computerized tomography. All *P* values were determined by two-sided Student’s *t* test. **b** *NCOA3* expressions in plasma cells from healthy controls (PC, *n* = 26), newly diagnosed MM patients (NDMM, *n* = 35), and refractory or relapse MM patients (RRMM, *n* = 30). Two-sided *P* values were determined by Student’s *t* test; mean ± SD. **c** *NCOA3* expression in newly diagnosed MM patients without known mutations (non-M, *n* = 67), with t(4;14) (*n* = 43) and other cytogenetic abnormalities (including t(11;14), t(16;14), and 1q21; *n* = 22). Two-sided *P* values were determined by Student’s *t* test; mean ± SD. **d** *NCOA3* expression in patients with complete response (CR, *n* = 12) and patients with refractory or relapse (RR, *n* = 12) before (pre-T) and after treatment (post-T), and (**e**) shows the difference of *NCOA3* expression in the two groups. Purple color, t(4;14)^+^. Two-sided *P* value was determined by Student’s *t* test; mean ± s.d. from *n* = 3 independent experiments. **f** The correlation coefficient between the *NCOA3* expression and numbers of bone lesion in MM patients (*n* = 3 independent experiments from *n* = 19 samples); *x*^2^ = 0.5806; *P* < 0.0001. **g** Immunohistochemical images of bone marrow biopsies from two patients with disease progression before (pre-T) and after treatment (post-T). Scale bar, 200 μm. **h** Correlation of *NCOA3* expression with progression-free survival (PFS) and overall survival (OS) in patients after receiving bortezomib (BTZ)-based treatment regimens in our cohort (*n* = 64). All *P*-values were determined by Pearson Coefficient and Log-ranks test. Source data are provided as a Source Data file.

To determine whether SRC-3 has clinical significance in patients with MM, we analyzed the correlation of SRC-3 expression with disease progression in MM patients. Among our cohort of 133 patients, 82 cases were newly diagnosed and 51 cases were refractory or relapsed (RR) MM. RR patients received either at least eight cycles of BTZ-based regimens for induction, or BTZ-based regimens for maintenance therapy before samples were collected; therefore, MM cells had at least one year of exposure to BTZ. All newly diagnosed patients were treated with BTZ-based regimens and their outcomes were categorized as complete response (CR) or RR accordingly^25^ (Table 1). Clinical evaluation for the establishment of a CR was reflected partially by bone-lesion restoration and a strikingly abrogated plasma-cell percentage post-treatment (post-T) compared with that at the pre-treatment (pre-T) stage in our study. We found that patients with a CR had concomitantly declined SRC-3 mRNA levels. However, in RR patients—whose bone lesions were deteriorated and plasma-cell percentages were augmented after at least eight cycles of BTZ-based regimens—SRC-3 mRNA levels were significantly increased (Fig. 1a). Moreover, this SRC-3 expression pattern was confirmed in plasma cells from both newly diagnosed MM patients and RR patients after BTZ-based regimens compared with those from healthy donors (Fig. 1b). Interestingly, SRC-3 expression was significantly correlated with a t(4;14) cytogenetic abnormality, but it was not significantly correlated with any other abnormalities such as t(11;14), t(16;14), and 1q21 (Fig. 1c). Among 12 CR patients, the percentage of bone marrow plasma cells (BMPC) were all suppressed under 5%, and in12 RR patients, the percentages were not significantly changes (Fig. S1a), we found SRC-3 expressions were all remarkably decreased in CR patients and increased in RR patients after BTZ-based treatments, respectively (Fig. 1d); the difference between CR and RR patients was significant (Fig. 1e), and their bone-lesion numbers were positively correlated with SRC-3 mRNA levels (Fig. 1f). However, although SRC-3 expression declined, no significant difference was observed between patients with partial response (PR) and very good partial response (VGPR) (Fig. S1b). Correspondingly, the protein level of SRC-3 was also obviously augmented in biopsies from patient with disease progression (Fig. 1g), and significantly higher in RR patients than in newly diagnosed MM patients (Fig. S1c, d). Our follow-up analysis indicated that SRC-3 expression was negatively correlated with both progression-free survival (PFS) and overall survival (OS); for SRC-3*-*high patients and SRC-3-low patients, the PFS medians were 15.5 months versus 28 months, respectively, and the OS medians were 64 months versus 91 months, respectively (Fig. 1h). Using an independent cohort of 542 MM patients (GSE2658), we also confirmed that high expression of SRC-3 was associated with significantly worse overall survival (*P* = 0.0198), although SRC-3 expression was only slightly increased in the active MM patients compared with normal plasma cells (NP) or monoclonal gammopathy of undetermined significance (MGUS) (Fig. S1e, f); in another cohort of 264 MM patients (GSE9782), SRC-3 expression was significantly lower in patients response to BTZ-based regimens than those without response (*P* = 0.0203) (Fig. S1g), and the OS and PFS were all remarkably extended in SRC-3 low patients compared with SRC-3 high patients (647 m vs. 300 m, 169 m vs. 119 m, respectively) (Fig. S1h, i). Collectively, these data suggested that SRC-3 plays a pivotal role in the treatment response and clinic outcomes of MM patients.

**Table 1:** Demographics and characteristics of patients enrolled in this study.

Characteristics	Total (*n* = 133)	NDMM (*n* = 82)	RRMM (*n* = 51)
Median age (range)	63 (37–87)	59 (37–79)	66 (46–87)
Gender			
Male	78 (58.6%)	47 (57.3%)	33 (64.7%)
Female	55 (41.4%)	35 (42.7%)	18 (35.3%)
Chromosomal abnormalities (%)^a^			
t (4;14)	22 (16.5%)	9 (10.9%)	13 (25.4)
t (11;14)	19 (14.3%)	10 (12.2%)	9 (10.9%)
t (14;16)	5 (3.75%)	3 (3.65%)	2 (3.92%)
del13q,del17p	50 (37.6%)	32 (39%)	18 (35.3%)
R-ISS stage			
I	35 (26.3%)	26 (31.7%)	9 (17.6%)
II	47 (35.3%)	27 (32.9%)	20 (39.2%)
III	51 (38s.3%)	19 (23.2%)	32 (62.7%)
Induction or maintenance regimen			
VD	11 (8.3%)	11 (13.4%)	0 (0)
VCD	93 (69.9%)	62 (75.6%)	31 (60.8%)
VTD	29 (21.8%)	9 (11%)	20 (39.2%)

*NDMM* newly diagnosed MM, *rrMM* refractory or relapsed MM, *BD* bortezomib, dexamethasone, *BCD* bortezomib, dexamethasone, cyclophosphamide, *VTD* bortezomib, thalidomide, dexamethasone

^a^The status of 1 patient is unknown

### SRC-3 levels are correlated with sensitivity to BTZ treatment in myeloma cells in vitro

**Fig. 2:**
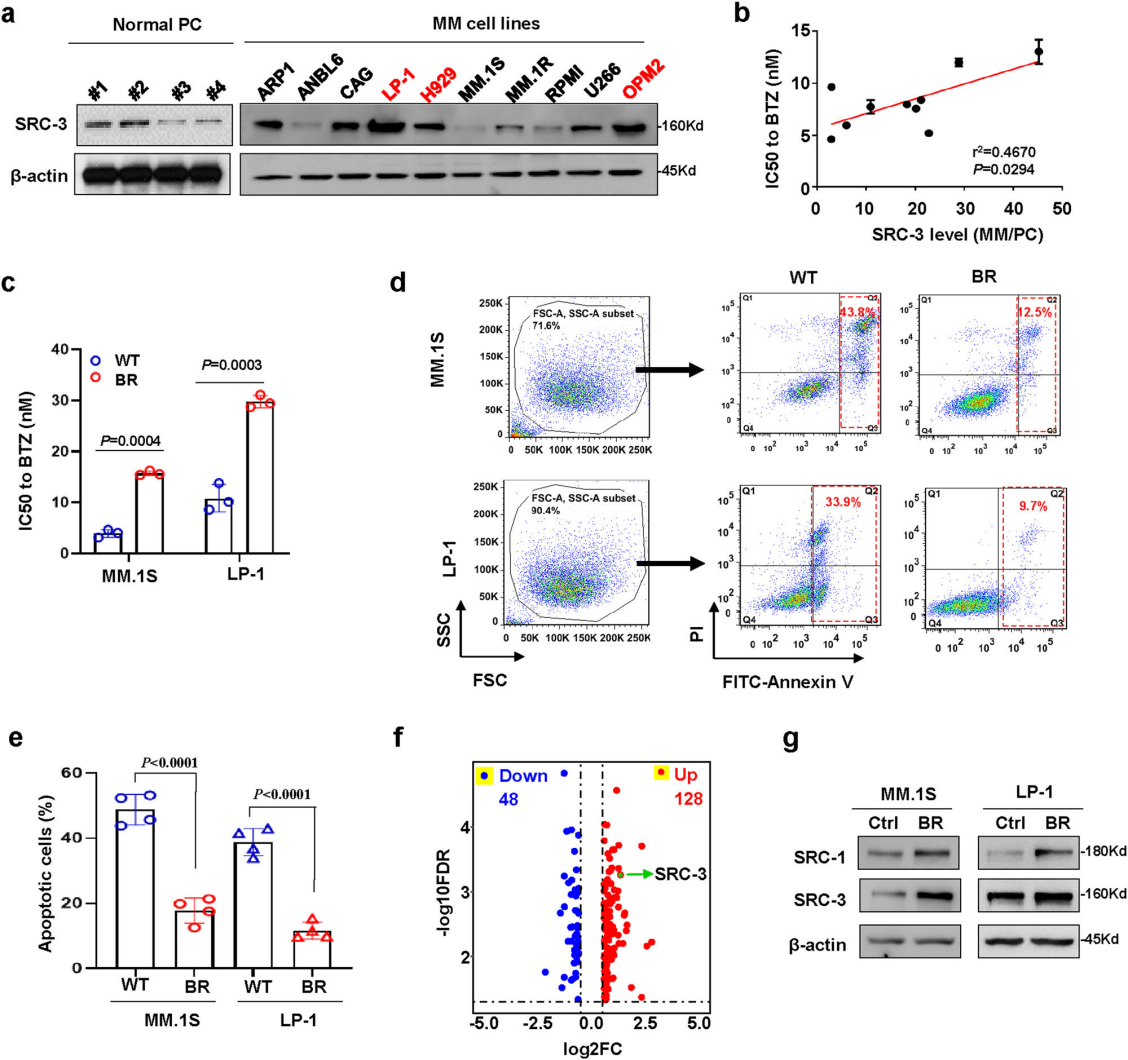
SRC-3 enhances drug resistance to proteasome inhibitor in myeloma cells. **a** Representative images (*n* = 3 biologically independent experiments) of Western blotting show SRC-3 levels in myeloma cell lines (*n* = 10) and plasma cells (PC, *n* = 4) from healthy controls. Red, cytogenetic t(4;14) cells. **b** Correlation coefficient between the SRC-3 levels and IC_50_ in myeloma cells (*n* = 3 independent experiments from *n* = 10 cell lines). *P* values of mean ± s.d. were determined by Pearson Coefficient. **c** Alteration of IC_50_ in bortezomib (BTZ)-resistant (BR) MM.1S and LP-1 cells compared to the parental wild type (WT) cells (*n* = 3 biologically independent experiments). Two-sided *P*-values were determined by Student’s *t* test; mean ± s.d. **d** Representative gating strategy of flow cytometry and apoptosis assay of the wild type (WT) and bortezomib (BTZ)-resistant (BR) MM cells induced by 5 nM BTZ for 48 h, and (**e**) analyzes the difference between two groups (*n* = 4 biologically independent experiments). Two-sided *P*-values were determined by Student’s *t* test; mean ± s.d. **f** Shown are 48 downregulated and 128 upregulated proteins (cutoff, 1.5 folds) in the bortezomib (BTZ)-resistant (BR) MM.1S cells compared to the wild type (WT) cells detected by Stable isotope labeling with amino acids in cell culture (SILAC) assay. **g** Elevated SRC-3 in wild type control (Ctrl) and bortezomib (BTZ)-resistant (BR) MM.1S and LP-1 cells by Western blotting (*n* = 3 biologically independent experiments). Source data are provided as a Source Data file.

We next examined SRC-3 levels in 10 myeloma cell lines with different genetic backgrounds^26^. We found that SRC-3 levels varied but were significantly higher in LP-1, H929, and OPM2 cells with t(4, 14) chromosomal abnormalities, but were similar across normal plasma cells (Fig. 2a). Our previous study has demonstrated that LP-1, H929, U266, and OPM-2 cells exhibited higher half-maximal inhibitory concentrations (IC_50_) to BTZ compared with those of other cell lines^27^; intriguingly, a significant positive correlation was found between BTZ-specific IC_50_ values and SRC-3 levels in these cell lines (Fig. 2b, Fig. S2a). Using our previously established BTZ-resistant myeloma cells^26^, for which their IC_50_ to BTZ was greatly augmented (Fig. 2c) and the anti-apoptotic capacity was remarkably enhanced (Fig. 2d, e), we detected proteomic alterations through Stable Isotope Labeling with Amino Acids in Cell culture (SILAC) assays. Indeed, we found SRC-3 in 128 increased proteins but not in 48 decreased proteins (fold change ≥1.5) (Fig. 2f). We also confirmed SRC-3 alterations, and to a lesser extent for SRC-1 alterations, by Western blotting (Fig. 2g, Fig. S2b). In addition, some other differently expressed proteins—such as several subtypes of HAL-DR (DRA, DRB1, DRB3)^28^, adhesion molecules (ITGA4, ITGB1)^29^, and ABC transporters—were found to be correlated with MM drug resistance (S Data 1).

**Fig. 3:**
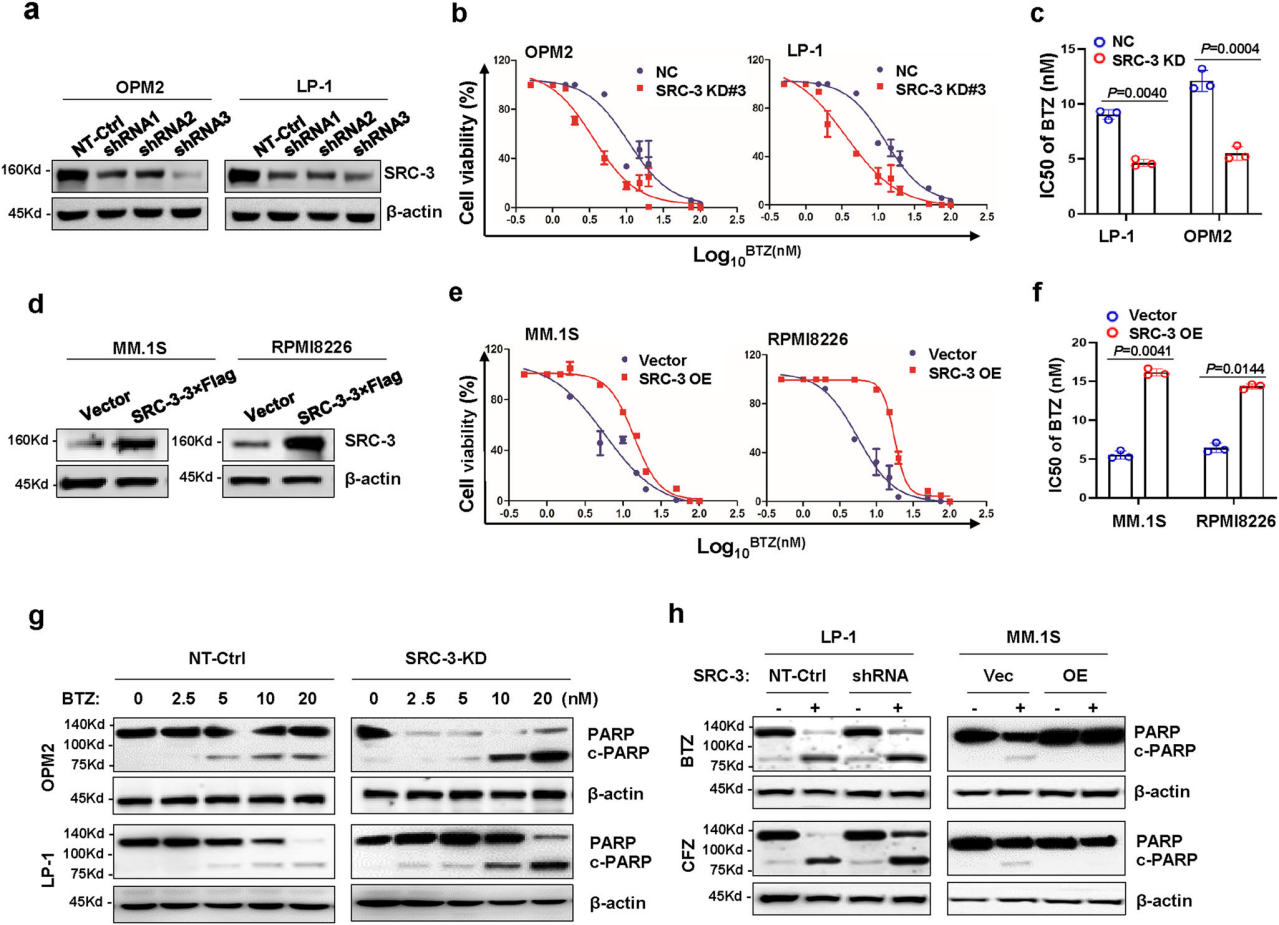
Manipulation of SRC-3 expression alters sensitivity to BTZ treatment in vitro. **a** Representative Western blotting (n = 3 biologically independent experiments) shows the knockdown effects in OPM2 and LP-1 cells infected with lentivirus carrying three shRNAs targeting three different coding sequencing of *NCOA3* gene (shRNA1, 2, 3) compared to the non-target control (NT Ctrl). **b** Alteration of IC_50_ to BTZ treatment in the NT Ctrl (NC) and SRC-3 knockdown (KD) cells (*n* = 3 biologically independent experiments; mean ± s.d.), and (**c**) comparison of the IC_50_ values of NT Ctrl (NC) and SRC-3 knockdown (KD) cells (*n* = 3 biologically independent experiments). Two-sided *P*-values were determined by Student’s *t* test; mean ± s.d. **d** Representative Western blot (n = 3 biologically independent experiments) shows the ectopic expression of SRC-3 in MM.1S and RPMI8226 cells infected with lentivirus carrying the SRC-3-3 × flag (SRC-3 OE) compared to the vector control (Vec). **e** Alteration of IC_50_ to bortezomib (BTZ) treatment in the vector control (Vec) and SRC-3 overexpression (OE) cells (*n* = 3 biologically independent experiments; mean ± s.d.). **f** Comparison of the IC_50_ value in the vector control and SRC-3 overexpression cells (*n* = 3 biologically independent experiments). Two-sided *P*-values were determined by Student’s *t*-test; mean ± s.d. (**g**) and (**h**) Cleavage of PARP as the apoptotic marker in myeloma cells with SRC-3 expression manipulated by shRNA or ectopic expressing vector, treated with increasing dosage of bortezomib (BTZ) and carfizomib (CFZ, 5 nM) for 48 h (*n* = 3 biologically independent experiments). Source data are provided as a Source Data file.

To further define the role of SRC-3 in MM drug resistance, we manipulated the expression of SRC-3 using short-hairpin RNA (shRNA) or an ectopically expressing vector in endogenously high- or low-SRC-3 cells, respectively. As expected, the expression levels of SRC-3 in OPM2 and LP-1 cells were successfully suppressed via shRNAs targeting *NCOA3* (Fig. 3a), and the IC_50_ values for BTZ were significantly suppressed by about 2 fold (Fig. 3b, c); on the other hand, ectopic expression of SRC-3 in MM.1S and RPMI8226 cells (Fig. 3d) remarkably augmented the IC_50_ value by about 3 fold (Fig. 3e, f). Furthermore, knockdown of SRC-3 sensitized OPM2 and LP-1 cells to BTZ treatment (Fig. 3g). In contrast, overexpression of SRC-3 abrogated BTZ-induced MM apoptosis, as evidenced by anabatic-cleaved PARP and following the use of another proteasome inhibitor, carfilzomib (CFZ) (Fig. 3h). Additionally, we found that manipulation of SRC-3 altered cellular proliferation in myeloma cells (Fig. S2c, d).

### NSD2 is recruited to and stabilizes SRC-3

**Fig. 4:**
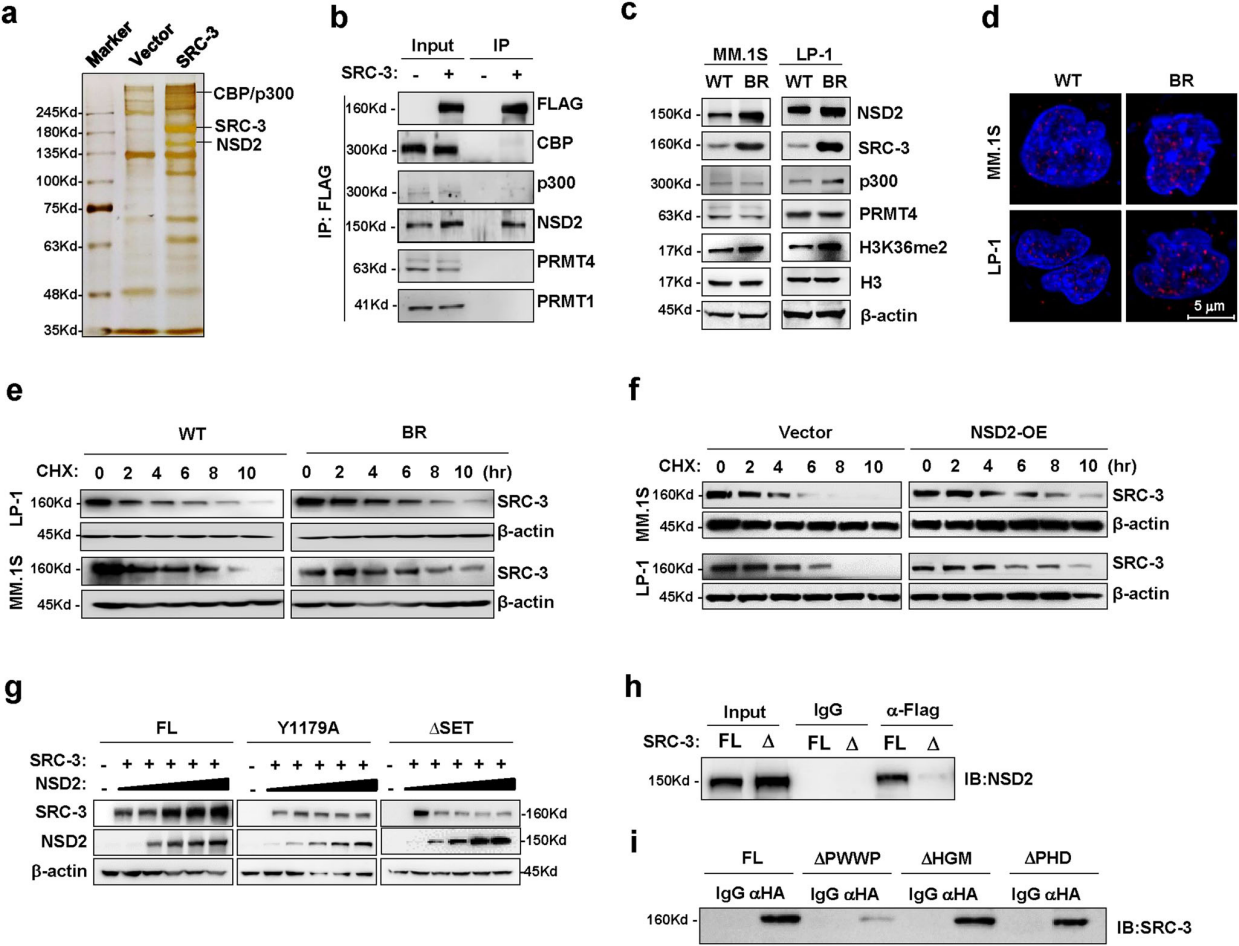
NSD2 is recruited to and contributes to SRC-3 stabilization. **a** Silver staining of LP-1 cells infected with lentivirus carrying SRC-3-3 × flag for 72 h (*n* = 3 biologically independent experiments). **b** Co-immunoprecipitation (Co-IP) assay shows interactions between CBP, p300, PRMT1, PRMT4, NSD2 and SRC-3. Input, 2% lysate (*n* = 3 biologically independent experiments). IP, M2-flag antibody. **c** Western blotting shows the alteration of SRC-3, NSD2, p300, PRMT4, H3K36me2 protein levels in the wild type (WT) and bortezomib (BTZ)-resistant (BR) MM.1S and LP-1 cells (*n* = 3 biologically independent experiments). **d** Proximity ligation assay (PLA) shows protein–protein interaction between NSD2 and SRC-3 (*n* = 30 cells from 3 biologically independent experiments). **e** Degradation rate of SRC-3 protein in WT and bortezomib (BTZ)-resistant (BR)-myeloma cells treated with 20 μM cycloheximide (CHX) for up to 10 h (*n* = 3 biologically independent experiments). **f** Degradation rate of SRC-3 protein in MM.1S and LP-1 cells infected with lentivirus carrying NSD2-HA (NSD2-OE) or vector control (Vector) for 72 h treated with 20 μM CHX for up to 10 h (*n* = 3 biologically independent experiments). **g** SRC-3-3 × flag levels in HEK293T cells co-transfected with full-length NSD2 (FL), SET domain depletion NSD2 (ΔSET), or Y1179A mutation NSD2 (Y1179A) for 48 h (*n* = 3 biologically independent experiments). **h** Immunoprecipitation assay for full-length SRC-3 (FL) or AD2-domain depletion SRC-3 (ΔAD2) in HEK293T cells (*n* = 3 biologically independent experiments). **i** Immunoprecipitation assay for full-length NSD2 (FL) or truncations with PWWP domain depletion (ΔPWWP), HGM domain depletion (ΔHGM), or PHD domain depletion (ΔPHD) with SRC-3 in HEK293T cells (*n* = 3 biologically independent experiments). Input, 2% whole lysate; IP, M2-flag antibody or HA antibody. Source data are provided as a Source Data file.

To further investigate how SRC-3 influences MM drug resistance, we ectopically overexpressed flag-tagged SRC-3 in MM.1S cells and immunoprecipitated the SRC-3 complex for mass spectrometry. In addition to previously reported partners (i.e., CBP/p300, PRMT1, and PRMT4), we discovered a protein in the SRC-3 complex, namely, histone-lysine N-methyltransferase NSD2 (Fig. 4a). Compared with CBP and p300, NSD2 exhibited a obviously stronger combination with SRC-3 compared with that of 2% input (Fig. 4b). Indeed, protein levels of NSD2 and SRC-3 were both elevated concomitantly with H3K36me2 in BTZ-resistant myeloma cells, independent of cytogenetic backgrounds, but this elevation was not observed in p300 or PRMT4 (Fig. 4c, Fig. S3a). As expected, NSD2 and SRC-3 interactions were detected remarkably augmented in the BR cells compared to the WT cells through proximity ligation assays (PLAs) (Fig. 4d, Fig. S3b), and immunofluorescent assays also indicated that they had identical subcellular localizations in cellular nuclei (Fig. S3c). Intriguingly, we found that the half-life of SRC-3 was significantly extended in BR myeloma cells (Fig. 4e, Fig. S4a); this finding was similar to what was observed in their parental cells with ectopic overexpression of NSD2 (Fig. S4b, Fig. 4f, Fig. S4c), and this protection occurred in a dose-dependent manner (Fig. S4d). When SRC-3 was co-expressed with full-length NSD2 or with a SET-domain depletion and a Y1179A mutant to induce loss of methyltransferase function in the SET domain (Fig. S4e), the protective effect was elicited only by full-length NSD2, but not following SET-domain mutations (Fig. 4g). Collectively, these previously undiscovered findings suggest that the methyl-transferase activity of NSD2 is essential for conferring the protective function of SRC-3.

To define the interaction domains of these two proteins, we constructed different truncations of SRC-3 and NSD2 for immunoprecipitation. We found full-length SRC-3, but not the AD2-domain-deleted truncation, was immunoprecipitated with NSD2 (Fig. 4h). In contrast, for NSD2 truncations with depletion of PWWP (ΔPWWP), HMG (ΔHMG), or PHD (ΔPHD) domains (Fig. S4f), we found that only depletion of the PWWP domain eliminated the interaction of NSD2 with SRC-3 (Fig. 4i), indicating that the AD2 domain of SRC-3 interacted with the PWWP domain of NSD2.

### Interaction with NSD2 promotes SRC-3 phase separation and alters the transcriptome in myeloma cells

**Fig. 5:**
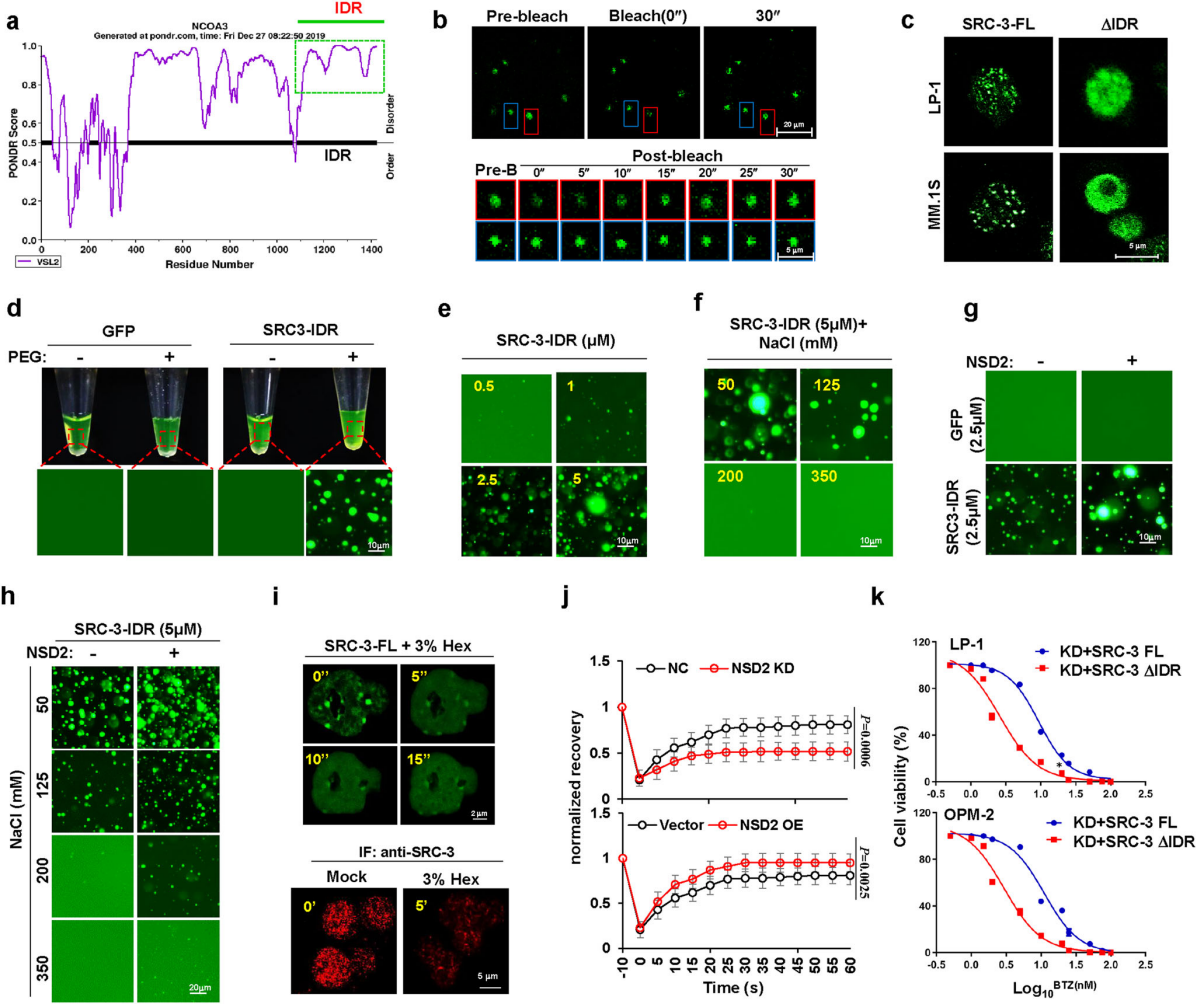
NSD2 enhances SRC-3 liquid–liquid phase separation in BRZ-resistant myeloma cells. **a** Prediction of intrinsically disordered regions (IDRs) (green frame) of SRC-3 protein using a PONDR algorithms. **b** Fluorescence recovery after photobleaching (FRAP) of a SRC-3-GFP focus (red framed) by 488 nm laser for 5″ bleaching and 30″ recovery in MM.1S cells, and the blue framed focus was used as a control (*n* = 3 biologically independent experiments). **c** Phase separation formation of full-length GFP-SRC-3 (FL) and IDR domain depletion GFP-SRC-3 (ΔIDR) in MM.1S and LP-1 cells (n = 3 biologically independent experiments). Scale bar, 5 μm. **d** Visualization of turbidity associated with droplet formation. Tubes containing SRC-3-IDR (right pair) or GFP (left pair) in the presence (+) or absence (−) of PEG-8000 (*n* = 3 biologically independent experiments). **e** Representative images (*n* = 3 biologically independent experiments) of droplet formation of GFP-SRC-3-IDR at different protein concentrations. **f** Representative images (*n* = 3 biologically independent experiments) of GFP-SRC-3-IDR at 5 μM protein concentration droplet formation at different salt concentrations. **g** Representative images (*n* = 3 biologically independent experiments) of droplet formation of GFP-vector or GFP-SRC-3-IDR in the presence (+) or absence (−) of NSD2. **h** Representative images (*n* = 3 biologically independent experiments) of droplet formation of GFP-vector or GFP-SRC-3-IDR in the presence (+) or absence (−) of NSD2 at different salt concentrations. GFP-vector or GFP-SRC-3-IDR was added to droplet formation buffer to achieve 5 μM protein concentration with a final NaCl concentration as indicated. **i** Phase separation formation of full-length GFP-SRC-3 (FL) in HEK293T cells before and after treatment with 3% 1,6-hexanediol at different times (upper). Representative micrographs (*n* = 3 biologically independent experiments) of confocal immunofluorescence for endogenous SRC3 droplet foci in MM.1S cells before and after treatment with 3% 1,6-hexanediol for 5 s (lower). **j** Kinetic recovery times of bleached GFP-tagged SRC-3 droplet foci in HEK293T co-transfected with NSD2 shRNA (NSD2 KD) or expressing vector (NSD2 OE) by 488 nm laser (*n* = 3 biologically independent experiments). Two-sided *P*-values of the comparisons between the final extent of recovery after photobleaching (i.e. 60 s)were performed using one-way ANOVA; mean ± s.d. **k** Alteration of IC_50_ to bortezomib (BTZ) treatment in LP-1 and OPM-2 SRC-3 knockdown (KD) cell lines in the presence of SRC-3 full length (SRC-3 FL) or IDR region depletion truncation (SRC-3 ΔIDR) (*n* = 3 biologically independent experiments). Source data are provided as a Source Data file.

Interestingly, when predicted the SRC-3 protein structure using three online-prediction algorithms to analyze whether SRC-3 exhibits liquid–liquid phase separation (LLPS) property (Fig. S5a, b), we found a putative intrinsically disordered region (IDR) with the highest strength among all candidates (average strength = 0.928; 1,085–1,424 amino acids) (Fig. 5a), which covers two AD domains responsible for protein–protein interactions^30^. When endogenous SRC-3 proteins were detected using immunofluorescence, they were shown as discrete puncta rather than in a diffused status in fixed MM cells, suggesting a possible property of a protein when entering LLPS state (Fig. S5c). Moreover, such puncta were also observed in live MM cells when a GFP-SRC-3 fusion protein was expressed, and when it was bleached with a 488-nm laser, the bleached puncta were reassembled quickly (Fig. 5b, red-framed foci), and kinetic recovery of GFP-SRC-3 fluorescence showed that the majority of SRC-3 foci fluorescence recovered within 10 s (Fig. S5d). To test the importance of the IDR region for SRC-3 phase separation, we constructed GFP-fusion IDR proteins (Fig. S5e) and an IDR-depleted SRC-3 fusion protein to measure optoDroplet formation. Intriguingly, we found that only the full length of SRC-3 could format an aggregation, whereas depletion of the IDR domain resulted in a diffused formation of SRC-3 (Fig. 5c). Importantly, the GFP-SRC-3-IDR fusion protein formed droplets at a minimum concentration of 0.5 μM and the droplets were augmented in a dose-dependent manner in the presence of 10% PEG8000 in vitro (Fig. 5d, e, Fig. S5f); moreover, this aggregation was abolished by increasing concentrations of NaCl (Fig. 5f). To clarify whether NSD2 is pivotal to SRC-3 LLPS or only protects SRC-3 protein from degradation, we added purified NSD2 into the GFP-SRC-3-IDR system and observed markedly augmented droplets (Fig. 5g), remarkably enhanced LLPS formation at lower concentration (Fig. S5g), and obviously postponed abolishment of GFP-SRC-3-IDR LLPS treated with NaCl (Fig. 5h). We also found the treatment of 1,6-hexanediol caused a reduction in the formation of exogenously expressed GFP-SRC-3 puncta in HEK293T cells, as well as in endogenous SRC-3 puncta in MM.1S cells (Fig. 5i). Akin to the in vitro results, when NSD2 was depleted in myeloma cells (Fig. S5h), the reassembly of GFP-SRC-3 FL was significantly inhibited, whereas it was enhanced when NSD2 was overexpressed in vivo (Fig. 5j). However, when the SET domain of NSD2 was depleted, it cannot be recruited into SRC-3 droplets, no obvious effect was observed in the recovery of SRC-3 aggregation after bleaching, when compared either to the vector control or to the full-length NSD2 (Fig. S5i). Moreover, using parental-paired KMS11^+/+^ and KMS11^+/−^ MM cells (with or without ectopic NSD2 overexpression) (Fig. S6a), we also observed that SRC-3 foci were significantly attenuated in KMS11^+/−^ cells (Fig. S6b, c). When the NSD2 expression was rescued in KMS11^+/−^ cells (Fig. S6d), the aggregation of SRC-3 was reinstated as well (Fig. S6e, f). Collectively, these data strongly suggest that SRC-3 possesses the property of LLPS, and it is suggestively correlated with NSD2 in MM cells.

To validate the role of the LLPS of SRC-3 in MM drug resistance, we first investigated the abundance of endogenous SRC-3 protein in WT and BR-MM cells, and found that SRC-3 foci were significantly abundant in BR MM cells (Fig. S6g, h). To clarify whether SRC-3 abundance or LLPS was correlated with MM drug resistance, we expressed ectopic SRC-3 full length (FL) or SRC-3 ΔIDR in OPM-2 and LP-1 cells with endogenous SRC-3 depletion and found that only the FL SRC-3 rescued the resistance to BTZ treatment (Fig. 5k, Fig. S6i). Notably, overexpression of SRC-3 FL completely prevented this rescue, whereas SRC-3 ΔIDR yielded even more cleavage of PARP compared with that of the control (Fig. S6j). Taken together, these results suggested that the LLPS of SRC-3 plays a critical role in MM drug resistance.

### SI-2 abolishes the phase separation of SRC-3 by abrogating its interaction with NSD2

**Fig. 6:**
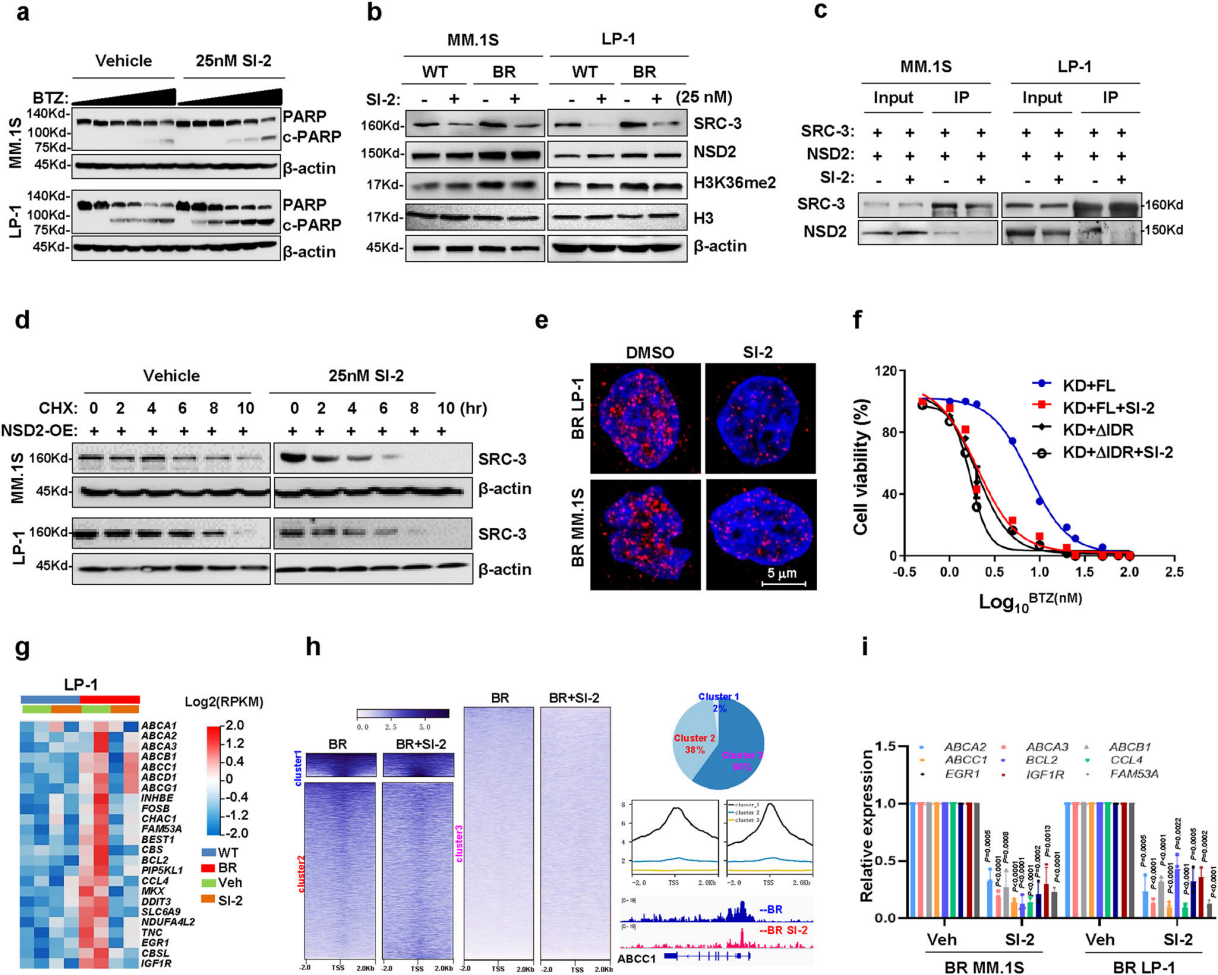
SRC-3 inhibitor SI-2 abolishes NSD2/SRC-3 interaction and sensitizes BTZ treatment in myeloma cells. **a** Cleavage of PARP as the marker of apoptosis in MM cell treated with bortezomib (BTZ) (0–20 nM) in the presence or absence of 25 nM SI-2 for 24 h (*n* = 3 biologically independent experiments). **b** Levels of SRC-3, NSD2, H3K36me2 in wild type (WT) and bortezomib (BTZ)-resistant (BR) myeloma cells treated with 25 nM of SI-2 for 24 h (*n* = 3 biologically independent experiments). **c** Co-IP assay shows interaction between SRC-3 and NSD2 in myeloma cells treated with 25 nM SI-2 for 16 h (*n* = 3 biologically independent experiments). Input, 2% whole cell lysate. IP, anti-SRC-3 antibody. **d** Degradation rate of SRC-3 protein in myeloma cells stably expressing NSD2 treated with 20 μM cycloheximide (CHX) for up to 10 h with or without 25 nM SI-2 (*n* = 3 biologically independent experiments). **e** Confocal immunofluorescence for endogenous SRC-3 droplet foci in bortezomib (BTZ)-resistant (BR) myeloma cells treated with 25 nM SI-2 or vehicle for 24 h (*n* = 3 biologically independent experiments). Scale bar, 5 μm. **f** Alteration of IC_50_ to bortezomib (BTZ) treatment in LP-1 SRC-3-KD cells treated with 25 nM SI-2 or vehicle for 24 h in the presence of SRC-3 full length (FL) or IDR region depletion truncation (ΔIDR) (*n* = 3 biologically independent experiments). **g** Heat map for drug resistance responsible genes alteration in 25 nM SI-2 or vehicle-treated bortezomib (BTZ)-resistant (BR) myeloma cells (*n* = 2 biologically independent experiments). **h** ChIP-seq profile for 3 clusters of genes enriched by H3K36me2 in bortezomib (BTZ)-resistant (BR) LP-1 cells treated with DMSO or 25 nM of SI-2 (*n* = 3 biologically independent experiments). Pie chart, percentage of 3 clusters of genes enriched by H3K36me2 in two groups; Density plots, changes of H3K36me2 density around the transcription start site (TSS) region of genes in 3 clusters. **i** qPCR shows the selected gene expressions in bortezomib (BTZ)-resistant (BR) myeloma cells treated with 25 nM SI-2 for 24 h. (*n* = 3 biologically independent experiments). Two-sided *P*-values were determined by Student’s *t* test; mean ± s.d. Source data are provided as a Source Data file.

To determine the translational significance of targeting SRC-3 in overcoming MM drug resistance, we examined the effect of a SRC-3 inhibitor, SI-2^31^. The combination of SI-2 at 25 nM significantly improved the sensitivity to BTZ treatment and partially improved the sensitivities to melphalan and lenalidomide treatments (Fig. 6a, Fig. S7a). Administration of SI-2 alone, however, did not induce apoptosis in myeloma cells, even at 200 nM, as no cleaved caspase-3 was observed (Fig. S7b). Remarkably, SI-2 treatment dramatically reduced SRC-3 levels in BR-MM cells and also moderately inhibited H3K36me2 levels (Fig. 6b), but had no obvious effect on other partners, such as NSD2, P300, PRMT1, PRMT4, or H3K27Ac levels (Fig. S7c). To explore whether downregulation of SRC-3 is dependent on SRC-3/NSD2 interactions, we implemented co-immunoprecipitation (Co-IP) experiments with or without SI-2 for a shorter incubation time (16 h) to exclude the impact of SRC-3 degradation (Fig. S7d); we found that the interaction of SRC-3 with NSD2 was almost entirely eliminated in the presence of SI-2 (Fig. 6c). In particular, the protective effect of NSD2 on SRC-3 protein stabilization was attenuated in the presence of SI-2, as the half-life of SRC-3 was dramatically shortened (Fig. 6d, Fig. S7e). As a consequence, the SRC-3 aggregation in BR MM cells was significantly diminished (Fig. 6e, Fig. S7f). To clarify whether SI-2 sensitizes MM cells to BTZ treatment by hindering SRC-3 LLPS, we treated endogenous SRC-3 KD LP-1 cells that were rescued by ectopic SRC-3 FL or ΔIDR overexpression with 25 nM of SI-2 and increasing dosages of BTZ. We found that SI-2 abolished the recovered resistance in FL-rescued cells, but it had only a limited effect in ΔIDR-rescued cells (Fig. 6f, Fig. S7g). Collectively, these results suggested that SI-2 eliminates the SRC-3 function by disrupting the direct binding of NSD2 at the IDR domain of SRC-3.

To elucidate transcriptomic features of SRC-3 that may be responsible for drug resistance, we employed RNA sequencing (RNA-seq) to define networks of genes in SI-2-treated myeloma cells. This RNA-seq analysis revealed that an ample number of genes were significantly upregulated in BR-MM cells, including anti-apoptotic genes such as ATP-binding cassette transporters (ABC transporters), *BCL2*, and *CCL4*, as well as direct SRC-3-downstream targets, such as *CBS, TNC, EGR1*, and *IGF1R* (Fig. 6g, S Data 2). Chromatin-immunoprecipitation sequencing (ChIP-seq) revealed that the recruitment of H3K36me2 on transcription start sites (TSS) of some genes that could be distinguished into three patterns; furthermore, SI-2 exposure reduced H3K36me2 recruitment on genes governing apoptosis, such as *ABCC1*, which hinders gene-transcription elongation through RNA polymerase II (Fig. 6h, S Data 3). Additionally, abolishment of increased H3K36me2 modifications on the TSS of SRC-3 target genes was confirmed by ChIP-PCR using H3K36me2 or NSD2 antibodies (Fig. S7h) and quantitative PCR (qPCR) (Fig. 6i). Together, these findings revealed that pharmacologically targeting SRC-3 through SI-2, at least in part, abrogates transcriptional alterations in myeloma cells responsible for drug resistance.

### SI-2 overcomes BTZ resistance and restores myeloma-induced bone lesions in vivo

**Fig. 7:**
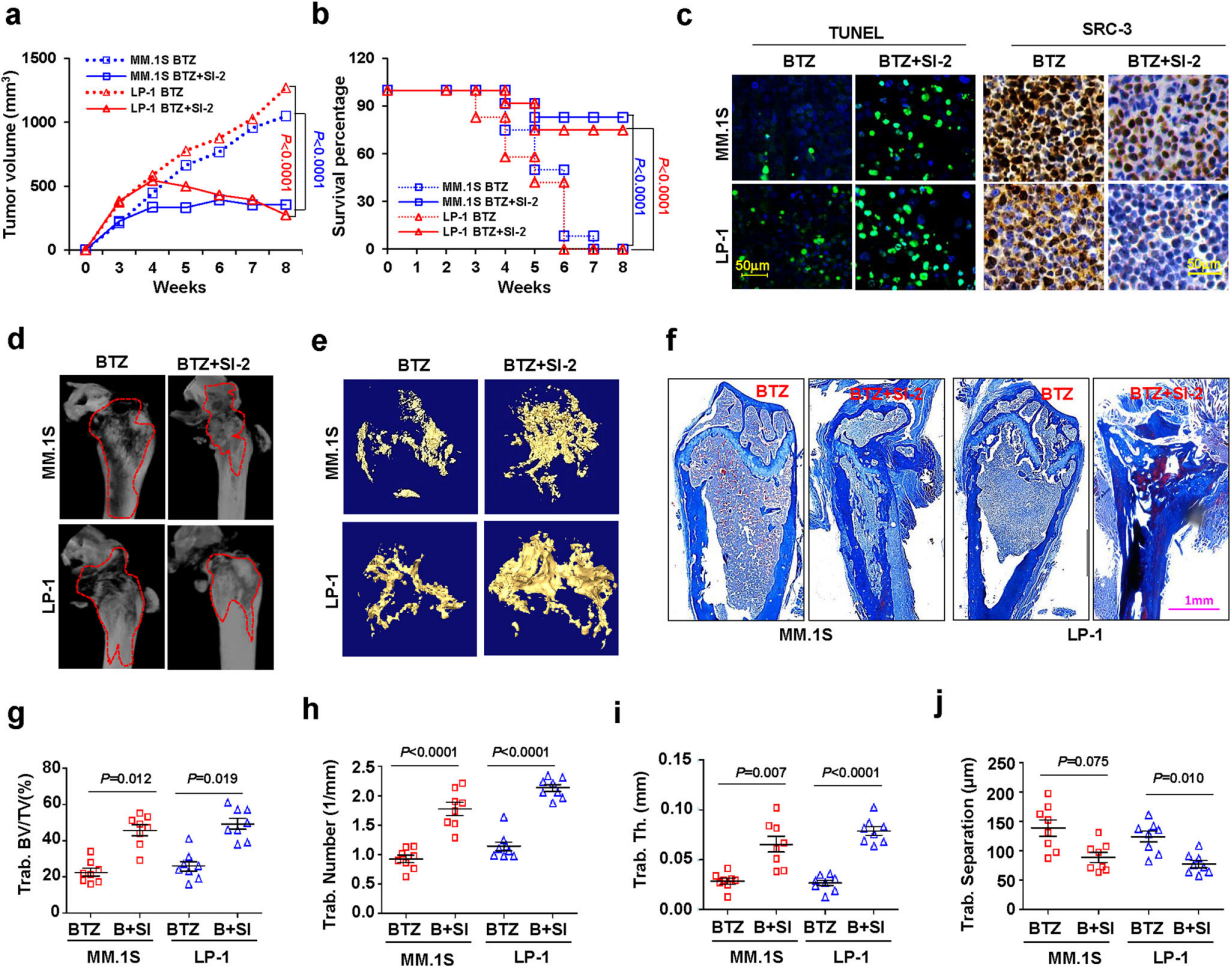
SI-2 abolishes BTZ drug resistance and rehabilitates bone disruption in vivo. **a** Tumor growth of bortezomib (BTZ)-resistant (BR) MM.1S or LP-1 cells (3 × 10^6^ cells/mouse) in NSG mice combined treatment with SI-2 and BTZ (*n* = 12 mice per group). Two-sided *P*-values were determined by two-way ANOVA test; mean ± s.d.; red *P* for LP-1 and blue *P* for MM.1S. **b** Survival rate of mice at the time point of tumor size = 15 mm^3^ (*n* = 12 mice per group). Two-sided *P*-values were analyzed using log-rank test; red *P* for LP-1 and blue *P* for MM.1S. **c** Representative micrographs (*n* = 12 biologically independent samples) of immunohistochemistry staining in tissues from xenograft of mice treated with bortezomib (BTZ) or BTZ plus SI-2 to detect SRC-3 level and apoptosis using TUNEL kit. Scale bar, 50 μm. **d** Representative microCT reconstructions of mouse femurs bearing bortezomib (BTZ)-resistant (BR) MM.1S and LP-1 cells (5 × 10^5^/mouse) and treated with BTZ (0.5 mg/kg) or BTZ + SI-2 (2 mg/kg SI-2) (n = 8 mice per group). **e** 3D reconstructions of bone trabecula in metaphyseal regions (*n* = 8 mice per group). **f** Masson staining shows the histological disruption of mice femur (*n* = 8 biologically independent samples). Measurement of (**g**) the percentage of bone volume to total volume (BV/TV), (**h**) trabecular number, (**i**) trabecular thickness, and (**j**) trabecular separation in the metaphyseal regions of the mice femurs in the bortezomib (BTZ) and BTZ + SI-2 groups were analyzed (n = 8 mice per group). Two-sided *P* values were determined by Student’s *t*-test; mean ± s.d. Source data are provided as a Source Data file.

To evaluate the effects of SI-2 on remission of myeloma tumor growth and lytic bone lesions following BTZ in vivo, we established WT-MM-originated or BR-MM-originated xenografts or bone-lesion models, respectively, in NOD-*scid* IL2Rg^null^ mice^26^. We used SRC-3-low MM.1S cells and SRC-3-high LP-1 cells to evaluate whether anti-MM effects are mediated by targeting the SRC-3 protein. SI-2 administration alone had no obvious effects on tumor growth in WT-MM-derived models, nor did BTZ administration alone in BR-MM-derived models. We found significantly beneficial antitumor effects, however, using a combination of BTZ and SI-2 in both MM-derived xenografts, with an even more obvious efficacy in LP-1 cells than in MM.1S cells (Fig. S8a). Notably, we did not observe any significant toxic effects in mice from these manipulations, as there were no detected changes in body weight or bone metabolism (Fig. S8b, c). Interestingly, when tumors were derived from BR-MM cells, the combined treatment with SI-2 and BTZ remarkably suppressed or even halted tumor growth in most cases (Fig. 7a) and, moreover, significantly improved mouse survival (Fig. 7b). We also found significantly inhibited SRC-3 level, increased apoptotic cells, and halted cellular proliferation in the SI-2-treated groups (Fig. 7c, Fig. S8d); additionally, SI-2 treatment abrogated the elevated genes responsible for drug resistance in MM (Fig. S8e).

In terms of our bone-lesion models, for mice femurs bearing WT-MM cells, the combination of SI-2 with BTZ significantly alleviated bone disruption compared with that of BTZ alone, whereas SI-2 alone had no beneficial effect (Fig. S8f, g). In the femurs of mice bearing BR-MM cells, remission of bone disruption in the SI-2/BTZ combination group was observed by the shrinkage of the osteolytic lesion area and less cortical perforations upon treatment (Fig. 7d). Three-dimensional reconstruction of bone-trabecula measurements illustrated a well-preserved trabecular network at metaphyseal and diaphyseal regions of mouse femurs in the SI-2/BTZ combination group (Fig. 7e). Histologically, an attenuated disruption of bone trabecula was also visualized in the SI-2/BTZ combination group (Fig. 7f, Fig. S8h). Quantitative analysis of bone structure revealed a significant recuperation of trabecula disruption in the SI-2/BTZ combination group, with higher percentages of BV/TV (Fig. 7g), trabecula numbers (Fig. 7h), and cortical thickness (Fig. 7i), as well as a smaller size of trabecula separation at the metaphysis and diaphysis (Fig. 7j). Taken together, these in vivo phenotypes and biological data revealed that pharmacological targeting of SRC-3 abrogates BTZ-induced drug resistance and stimulates bone-lesion recovery in mice.

## Discussion

In the present study, we elucidated an important role of SRC-3 in proteasome-inhibitor-induced drug resistance in MM patients and immortalized cell lines. We found that SRC-3 mechanistically exerted this role through interactions with the histone methyltransferase NSD2 to stabilize SRC-3 and form liquid–liquid phase separation, consequentially remodeling chromatin and producing an adaptive transcriptome favoring myeloma-cell survival and disease progression. Notably, our findings also revealed significant anti-MM effects of a newly developed SRC-3 inhibitor, SI-2, with BTZ, and shed light on developing strategies using SI-2 to overcome drug resistance in MM patients.

Previous findings revealed that oncogenic SRC-3 is dominantly expressed in human CD19^−^ B cells, B lymphoblasts, and dendritic cells^32^. CD19^−^ B cells mainly consist of plasma cells (with only a few numbers of pro-B cells), from which MM originates^33^. Functional roles of SRC-1 and SRC-2 are mostly reported in tumor migration and metastasis, but SRC-3 also plays more important roles in tumor initiation, progression, and chemoresistance^34^. We found that SRC-3 expression was elevated more in RR patients than in newly diagnosed MM patients, as well as in patients with disease progression; moreover, correlational analysis showed a significant negative correlation of SRC-3 with clinical outcomes, confirming the role of SRC-3 in tumor progression that previously has been reported by other groups. Our SILAC assay showed that SRC-3 is one of the DEP proteins in BTZ-resistant MM cells, and that the other down- or upregulated proteins likely also have close relationships with SRC-3. Another contribution of our present study in regard to elucidation of SRC-3 in MM chemoresistance is that elevated SRC-3 in MM may be induced specifically by proteasome inhibitors, because RR patients in our study were treated with several rounds of BTZ-based regimens. Furthermore, our in vitro studies also verified that depletion of SRC-3 either by RNA interference (RNAi) or small-molecule inhibitors sensitized the anti-MM efficacy of BTZ. Thus, a variety of assays in our present study collectively revealed an important role of SRC-3 in acquired drug resistance in MM.

SRC-3 has been reported to be modified by SUMOylation for degradation^35^, and BTZ is a type of proteasome inhibitor that disturbs the proteasome-dependent protein degradation system. Therefore, modification of SRC-3 protein levels, or interactions with other protein complexes, may play important roles in SRC-3-mediated proteasome-inhibitor-induced drug resistance in MM. SRC-3 has been found to promote hormone-dependent cancers by coactivating nuclear receptors as well as transcription factors^18^. Four conserved structural domains constitute a platform for these coregulators to bind and execute chromatin remodeling and transcriptional regulation. Specifically, AD2 is the domain in which the histone methyltransferases, CARM1 and PRMT1, are reported to bind and promote histone methylation that subsequently facilitates chromatin remodeling^36^. In our present study, we discovered that NSD2 levels—but not p300, CARM1, or PRMT1 levels—were upregulated under BTZ treatment. A recent study has reported that NSD2 interacts with and methylates the non-histone protein, aurora kinase A, and promotes solid tumor cell proliferation^17^. Our present study also suggested that methyltransferase activity of NDS2 is essential for SRC-3 stability, because either depletion of the SET domain with methyltransferase activity or mutation of the key amino acid in the SET domain of NSD2 extinguished the NDS2-mediated protective effects on SRC-3. However, whether NSD2 regulates SRC-3 expression at the transcriptional level was not investigated in the current study.

One consequence of protein–protein interactions and modifications is to form LLPS, often through intrinsically disordered protein sequences^37^. We identified that SRC-3, but not NSD2, had the capacity to form LLPS, and it is closely correlated with the NSD2 in MM cells. The functional consequences of phase-separated condensates are involved in various biological activities, including modulating chromatin structure, gene expression, protein degradation, and signaling transduction.^38,39^ These functional consequences are particularly interesting when they occur in the nucleus, because the nuclear phase is reported to be critical for direct interaction and regulation of the transcriptome^38,40^. Importantly, altered LLPS usually leads to dysregulation of nuclear events or epigenetics, and consequentially causes tumorigenesis or progression, because condensation of proteins can exert augmented biological functions compared with those during a diffused status^41^. Besides, the destruction of SRC-3 LLPS also causes diffusion of SRC-3 and alters its normal localization in the nucleus, thus attenuating SRC-3 mediated gene transcription activity and likely other biological functions as well. An updated literature has indicated that small-molecule therapeutics in nuclear condensates could influence drug activity and drug resistance in cancer cells, thus optimize condensate partitioning may be valuable in developing improved therapeutics^42^. Our present study provided the first evidence that LLPS is involved in the drug resistance of MM. Specifically, we found that LLPS of SRC-3 enhanced recruitment of NSD2 to the condensate and exerted higher methyltransferase activity, leading to H3K36me2 modifications on the genes responsible for cellular survival and anti-apoptosis (e.g., ABC family genes and *BCL2*), which may promote resistance to chemotherapies. Thus, targeting the interaction of SRC-3 and NSD2, either by manipulating gene expression or using small-molecular inhibitors, may suppress LLPS formation and reduce NSD2-mediated H3K36me2 modifications without depleting NSD2 expression levels. Collectively, our present findings provide at least two mechanistic insights into the roles of SRC-3 in MM drug resistance, and also provide a perspective for better understanding how SRC-3 bridges chromatin modifications with transcriptional alterations to promote chemoresistance in MM.

Recently, newly developed small-molecule inhibitors for disrupting protein–protein interactions to eliminate activities or functions of target proteins have shed light on ways to exploit strategies for cancer therapy. In the present study, we also examined the efficacy of a SRC-3 inhibitor, SI-2, on overcoming resistance to BTZ therapy in myeloma cells. SI-2 has been verified to significantly inhibit breast cancer growth through a direct interaction with SRC-3, as well as to selectively reduce protein concentrations and transcriptional activities of SRC-3 without greatly affected cellular viability^31^. In addition, SI-2 also has been shown to exert a suppressive effect on lung tumor initiation by eliminating cancer stem-like cells^43^. Notably, Song et al. have reported that administration of SI-2 (at 2 mg/kg) has no observable acute or chronic toxicity in vivo^31^. In MM, we found that administration of SI-2 alone did not kill myeloma cells even at a high concentration, but it did sensitize the anti-MM efficacy of BTZ at nanomolar levels. We also found that SI-2 disrupted the interaction of SRC-3 with NSD2, and facilitated degradation of SRC-3. It is conceivable that SI-2 significantly affects drug-responsible genes and consequently influences cells that are prone to survival. Most important, we found that the anti-MM efficacy of SI-2 became more obvious in MM cells with t(4;14) translocations, in which NSD2 levels were predominantly high. Thus, our present findings highlight a possible translational application of SRC-3 inhibitors, such as SI-2, for clinical use in treating RR-MM patients.

In summary, our present findings elucidated the importance of SRC-3 in proteasome-inhibitor-induced drug resistance in myeloma cells, and uncovered its interaction with the histone methyltransferase NSD2. Our results also shed light on guiding the development of therapeutic strategies using the SRC-3 inhibitor, SI-2, for MM patients with t(4;14) translocations or induced RR from proteasome-inhibitor therapy.

## Methods

### Patient and ethics approvals

Diagnosis of newly diagnosed or relapsed/refractory MM patients by the International Myeloma Working Group (IMWG) criteria^44^, management regimens and response evaluation^25^, the Revised International Staging System (R-ISS) for MM^45^, and the key exclusion criteria^46^, were adopted accordingly. Briefly, patients of standard risk were induced with BTZ-based regimen (≥8 cycles) before maintenance, and patients of high risk were maintained with BTZ-based regimens; response to treatment regimens were evaluated after receiving 2 cycles of treatment, and both the primary and secondary refractory as well as relapsed patients were included; the bone disruptions were evaluated one year after the treatment. Bone marrow biopsies were taken without washout period. Preparation of CD138^+^ cells from MM patients and healthy controls have been described in a previous study^26^. This study was approved by the Ethics Committee of Tianjin Medical University, and all protocols conformed to the Ethical Guidelines of the World Medical Association Declaration of Helsinki. Signed informed consent was obtained from each participating individual prior to participation in the study.

### MM cell lines

Myeloma-cell lines MM.1R and U266 were purchased from ATCC (American Type Culture Collection, Manassas, VA, USA), RPMI-8226 and MM.1S from the National Infrastructure of Cell Line Resource (Beijing, China), NCI-H929 from Chinese Academy of Sciences Cell Bank (Shanghai, China), respectively; ANBL-6, ARP-1, CAG, OPM-2 were kind gifts from Dr. Robert Z. Orlowski at the Department of Lymphoma and Myeloma, UT MD Anderson Cancer Center. MM cells were cultured in RPMI-1640 media supplemented with 15% of fetal bovine serum, 100 U/ml of penicillin, 100 mg/ml of streptomycin, and 2 mM L-glutamine (Gibco, Life Technologies, Carlsbad, CA, USA). The HEK293T cells were cultured in DMEM-high glucose media with 10% fetal bovine serum, 100 U/ml of penicillin, 100 mg/ml of streptomycin, and 2 mM L-glutamine. These cells were all cultured at 37 °C in a humidified incubator with 5% CO_2_ (Gibco, Life Technologies, Carlsbad, CA, USA). All cells were STR authenticated (Biowing Biotech, Shanghai, China) and mycoplasma-free confirmed with the Universal Mycoplasma Detection Kit (ATCC, Manassas, VA, USA).

### Stable isotope labeling using amino acids in cell culture (SILAC) assay

The cells were labeled with either “heavy isotopic amino acids” (L-^13^C_6_-Lysine/L-^13^C_6_^15^N_4_-Arginine) or “light isotopic amino acids” (L-Lysine/L-Arginine) using a SILAC Protein Quantitation Kit (Pierce, ThermoFisher, Carlsbad, CA, USA) according to the manufacturer’s instructions. The cell line was grown for more than six generations before being harvested, to achieve more than 97% labeling efficiency. After that, the cells were further expanded in SILAC media to desired the cell number. Finally, the cells were washed with cold PBS two times and stored at −80 °C for further LC-MS/MS and bioinformatics analysis (PTM biolabs, Hangzhou, China).

### Transfection, virus package, and infection

Transient transfections to HEK293T cells were performed using polyethyleneimine (PEI) (Polysciences, Warrington, PA, USA) in the OPTI-MEM medium (Life Technologies, Carlsbad, CA, USA) with a ratio of 1:4 to 1:6 of DNA:PEI. Transient transfections to MM cells were performed using the Neon electroporation Transfection System (Invitrogen of Life Technologies, Carlsbad, CA, USA) according to the manufacturer’s instructions as previously reported^27^. Viral particles were produced by HEK293T cells in a 10 cm dish transfected with 4 μg PMD2G and 6 μg PSPAX2 packaging plasmids (Addgene, Watertown, MA, USA), together with 8 μg lentiviral expressing vectors encoding target genes listed in the S Table 1. Supernatant carrying the viral particles was harvested after transfection and concentrated to 1/100 volume by Poly (ethylene glycol) 8,000 (Sigma-Aldrich, St. Louis, MS, USA). For viral infection, 1 × 10^6^ myeloma cells were seeded and then added 50 μL viral concentration and 8 μg/mL polybrene, and cells were spun at 200 × *g* for 45 min at 20 °C. 12 h after infection, the medium was changed and cells were cultured for another 48 h until further management.

### Cell proliferation assay

Myeloma cells (5 × 10^3^) were treated as designed in 96-well plates for an appropriate time and then added 20 μL of CellTiter 96 AQueous One Solution Reagent (Promega, Fitchburg, WN, USA) into 100 μL of culture media, after incubation for 1–4 hr at 37 °C the plates were read at 490 nm with a Microplate Reader 550 (Bio-Rad Laboratories, Richmond, CA, USA). The following formula was used to calculate cell viability (%) = OD value of treatment group/OD value of control group ×100.

### Real-time PCR and RNA-seq experiment

Total RNA was isolated using Trizol (Life Technologies, South San Francisco, CA USA) according to the manufacturer’s instructions. Total RNA (2 μg) was reverse transcribed using the 5× All-In-One reverse transcription MasterMix (abm, Vancouver, Canada). Quantitative real-time PCR was performed by mixing cDNA, gene-specific primers, and EvaGreen 2× qPCR MasterMix (abm, Vancouver, Canada) in the QuantStudio 3 Real-Time PCR System (Applied Biosystems). Primer sequences are provided in the S Table 1. For RNA-sequencing assays, the quality of the purified RNA was tested on Agilent 2100 Bio analyzer (Agilent RNA 6000 Nano Kit) (BGI, Shenzhen, China). Libraries for cluster generation and DNA sequencing were prepared following an adapted method from the BGISEQ-500 platform. The low quality reads (more than 20% of the bases qualities are lower than 10) were filtered to get the clean reads. Then those clean reads were assembled into Unigenes, followed with Unigene functional annotation, SSR detection and calculation of the Unigene expression levels and SNPs of each sample. Finally, DEGs (differential expressed genes) were identified between samples and from clustering analysis and functional annotations.

### Flow cytometry analysis

MM cells were incubated in 12-well plates and administered with bortezomib with or without SRC-3 inhibitors. After incubation at 37 °C for the indicated time, apoptosis assay was then carried out using the Annexin V-FITC Apoptosis Detection Kit (Sigma-Aldrich, St. Louis, MO, USA) according to the manufacturer’s instructions. A total of 5 × 10^5^ cells were stained with 5 μL Annexin V-FITC and 1 μL of PI in the dark to ensure the population abundance. The cells were analyzed by a FACS Calibur instrument. The data of flow cytometry were analyzed with CellQuest 3.0 software (BD Biosciences, New Jersey, USA). Forward and side scatter gating strategy was used in flow cytometry analysis to identify the cells of interest based on the relative size and to remove debris and other events that are not of interest.

### Immunofluorescence staining (IF) and PLA

For IF assays, myeloma cells were fixed in 4% formaldehyde, then samples were permeabilized and blocked. After incubation with anti-SRC-3 antibody (1:500) (Cell Signaling Technology, #2126, Danvers, MA, USA) and anti-NSD2 antibody (1:250) (Abcam, ab75359, Cambridge, UK) overnight at 4 °C, samples were incubated with FITC-conjugated goat anti-rabbit IgG (1:2000) for 60 min at room temperature and nuclei were counterstained with DAPI. PLA assay was performed using Duolink In Situ (Sigma-Aldrich, St. Louis, MO, USA) according to the manufacturer’s instructions. Briefly, MM cells were deposited on glass slides and pre-treated with respect to fixation, retrieval, and/or permeabilization. After blocking, and sequentially incubating with a mouse anti-NSD2 antibody (1:250) (Abcam, ab75359, Cambridge, UK) and a monoclonal rabbit anti-SRC-3 antibody (1:500) (Cell Signaling Technology, #2126, Danvers, MA, USA) overnight at 4 °C. Species-specific secondary antibodies linked to specific oligonucleotides were added, and the sample was incubated for an additional 60 min at 37 °C. Ligation of PLA probes was done by adding a solution containing ligase to the sample at 37 °C for 30 min. Finally, signal amplification took place by rolling circle amplification of ligated PLA probes at 37 °C for 100 min. Samples were stained with DAPI to visualize cell nuclei. The addition of isotype control immunoglobulins instead of primary antibodies was used as a negative control. Signal detection was carried out by fluorescence imaging performed using an Olympus FV1000 IX81-SIM Confocal Microscope (Olympus, Tokyo, Japan).

### Tunnel assay

Tunnel assay was performed using the DeadEnd™ Fluorometric TUNEL System (Promaga, Tokyo, Japan) according to the manufacturer’s instructions. Briefly, the deparaffinized and rehydrated tissue sections were incubated with proteinase K (20 µg/mL) for 8–10 min. Then they were washed and incubated with Equilibration Buffer for 5–10 min. The tissue sections were fixed with 4% methanol-free formaldehyde solution for 5 min. Then they were washed and incubated with rTdT incubation buffer at 37 °C for 60 min in the dark. The reactions were terminated by incubation with 2× SSC for 15 min at room temperature. The samples were washed and stained by propidium (1 µg/mL) in PBS for 15 min at room temperature in the dark. Then the samples were washed three times and analyzed by the Olympus FV1000 IX81-SIM Confocal Microscope (Olympus, Tokyo, Japan).

### Fluorescence recovery after photobleaching (FRAP)

MM cells were subjected to FRAP experiments with an Olympus FV1000 IX81-SIM Confocal Microscope (Olympus, Tokyo, Japan). Photobleaching was performed using tornado mode with the 488 nm laser at 100% laser power for 1–5 s. EGFP fluorescence recovery was monitored with 488 nm laser using the free-run mode at ~5 s intervals. Fluorescence of unbleached site in the same view was also monitored as the control. Signal was presented as the ratio relative to the fluorescence signal before photobleaching.

### In vitro droplet assay

For protein purification, plasmids containing the GFP-fused protein were transformed into BL21 cells, and a fresh bacterial colony with OD_600_ of 0.8–0.9 was induced by 1 mM IPTG at 16 °C overnight. Pallets were resuspended in GST lysis buffer (50 mM Tris-HCl pH 7.5, 100 mM NaCl) containing 1 mM dithiothreitol, 0.2 mM phenylmethylsulfonyl fluoride, 1% Triton-X100, complete protease inhibitor and sonicated (4 cycle of 30 s on, 30 s off). The lysate was cleared by centrifugation at 12,000 ×g for 20 min at 4 °C and added with NaCl to 500 mM. Then the supernatants were added to 500 μL glutathione agarose (Thermo Fisher, 16100) (prewashed in lysis buffer). Tubes containing this agarose lysate slurry were rotated at 4 °C overnight. Then the packed agarose washed twice with GST lysis buffer containing 500 mM NaCl and twice with GST lysis buffer. Protein was obtained by cleavage of Pierce^TM^ HRV 3 C protease and judged by coomassie stained gel. For droplet assay, protein was added to solutions at varying concentrations with indicated final salt and molecular crowder concentrations in Buffer A (50 mM Tris-HCl pH 7.5, 10% glycerol, 1 mM DTT). The protein solution was immediately loaded onto a homemade chamber comprising a glass slide with a coverslip attached by two parallel strips of double-sided tape. Imaging was obtained by the Olympus FV1000 IX81-SIM Confocal Microscope (Olympus, Tokyo, Japan).

### Western blotting and co-immunoprecipitation (Co-IP)

Protein lysates were prepared in RIPA-buffer supplemented with complete protease inhibitors (Roche, Indianapolis, IN, USA). Cell lysate (50 μg) was separated by electrophoresis on SDS-PAGE gel and transferred to nitrocellulose membranes (Pall Corporation, Washington, NY, USA). Membranes were blocked with 5% non-fat milk for 1 h at room temperature and probed overnight at 4 °C with specific antibodies. Membranes were washed and incubated with horseradish peroxidase-conjugated secondary antibodies at room temperature, and finally were visualized using an enhanced chemiluminescence system (Millipore, Los Angeles, CA USA). For Co-IP assay, cells were harvested and lysed by NP-40 lysis buffer (50 mM Tris-Hcl pH 7.4, 150 mM Nacl) supplemented with complete protease inhibitors (Roche, Indianapolis, IN, USA) on ice for 30 min. Co-IP for exogenous expressed proteins, supernatant was incubated with anti-FLAG M2 Affinity Gel (Sigma-Aldrich, St. Louis, MO, USA), for endogenous assays myeloma cells, the supernatant was incubated with 2 μg specific antibodies at 4 °C overnight with protein G dynabeads (ThermoFisher Scientific, Carlsbad, CA, USA). The next day, the pellet was washed four times with NP-40 lysis buffer, and then subjected to western blotting analysis using the anti-NSD2 or anti-SRC3 antibodies, respectively. Antibodies used in this study were listed in the Supplementary File. The representative Western blot images for at least three independent experiments shown in the figures have been cropped and auto contrasted. Quantifications of Western blots were analyzed using Image J Version 1.53c (National Institutes of Health).

### Sliver staining and mass spectrometry

Samples were harvested by Co-IP and resolved in the gel by Western blot. Sliver stain was performed according to Pierce™ Silver Stain Kit (Thermo, Waltham, MA, USA) manufacturer’s instructions. Briefly, the gel was washed twice in ultrapure water, fixed twice in 30% ethanol, and washed sequentially in 10% ethanol and ultrapure water. Then the gel was sensitized by sensitizer working solution for 1 min, stained in stain working solution for 30 min after washed with water. The gel was washed twice with ultrapure water for 20 s, developed with developer working solution for 2–3 min until bands appeared, stopped with 5% acetic acid for 10 min. The specific bands were selected for mass spectrum. The corresponding bands were further excised and subjected to mass spectrum assay as previously described^47^.

### Chromatin-immunoprecipitation (ChIP) and ChIP-sequencing (ChIP-seq)

4 × 10^7^ cells were cross-linked with 1% formaldehyde for 10 min at room temperature and then quenched by the addition of glycine (125 mM final concentration) for 5 min. The cells were harvested and resuspended in 1 ml ChIP lysis buffer (1% SDS, 10 mM EDTA, 50 mM Tris-Hcl, pH 8.0), then incubated on ice for 10 min. Chromatin was fragmented to 200–500 bp using 12 cycles of ultrasonication (SONICS, Newtown, CT, USA). For each IP, chromatin was immunoprecipitated with 2 μg of antibody in IP dilution buffer (1% Triton X-100, 2 mM EDTA, 150 mM NaCl, 20 mM Tris-HCl, pH 8.0) at 4 °C overnight. Chromatin was precleared for 2 hr each with protein G agarose beads (Cell Signaling Technology, Danvers, MA, USA) after immunoprecipitation. The immunoprecipitated material was washed, once in TSE I buffer (20 mM TrisHCl pH 8.0, 2 mM EDTA pH8.0, 150 mM NaCl, 1% Triton X-100, 0.1% SDS), once in TSE II buffer (20 mM TrisHCl pH 8.0, 2 mM EDTA pH8.0, 500 mM NaCl, 1% Triton X-100, 0.1% SDS), once in LiCl buffer (10 mM TrisHCl pH 8.0, 250 mM LiCl, 1% deoxycholic acid, 1% NP40) and once in TE buffer (10 mM Tris pH 8.0, 1 mM EDTA pH8.0) before elution in elution buffer (100 mM NaHCO3, 1% SDS). After beads were removed, samples were cross-linked overnight at 65 °C and DNA was isolated using QIAquick PCR Purification Kit (QIAGEN, MD, USA). Precipitated DNA was analyzed by PCR or high-throughput sequencing (Beijing Genomics Institute, Beijing, China). Antibodies used in this study were listed in the supplementary file.

### Immunohistochemistry

Deparaffinized tissue slides were blocked with 3% H_2_O_2_ solution and antigen were retrieved with 10 mM citrate buffer (pH 6.0). After blocking, appropriately diluted primary antibodies were added onto the slides and incubated in a humidified chamber at 4 °C overnight, and then appropriately diluted biotinylated secondary antibody was incubated at room temperature for 1 hr. DAB substrate solution (Dako, K5361) (freshly made just before use) was used to reveal the color of antibody staining. Nuclei were localized by heamtoxylin staining for 1–2 min before mounting and capture.

### NOD/SCID Mouse Xenograft and Intra-bone bone lesion models

Animal studies were approved by the Committee on Animal Research and Ethics of Tianjin Medical University, and all protocols conformed to the Guidelines for Ethical Conduct in the Care and Use of Nonhuman Animals in Research. 4–6 weeks old female NOD.*Cg-Prkdc*^*scid*^*Il2rg*^*tm1Wjl*^/SzJ mice were used to establish the xenograft (*n* = 12) and intra-bone injection models (*n* = 8) as previously reported^48,49^. After 2 weeks when tumor sizes were palpable (~5 mm diameter), BTZ (0.5 mg/kg) was administrated intraperitoneally with DMSO as vehicle control or SI-2 (2 mg/kg) three times per week, and tumor volumes were monitored every 3 days as 1/2(L*W^2^) mm, where the L presenting the length and W representing width of tumor. Measurements of tumor size were achieved by two independent technicians and the average was used to calculate the growth curve. After treatment 5 weeks, for xenografts experiments, mice were sacrificed and the tumor xenografts were collected for IHC and TUNEL assays. MicroCT was performed on mice femurs with a Skyscan 1172 microtomograph (BrukermicroCT, Kontich, Belgium). After segmentation, the 3D models were constructed from the stack of 2D images with a surface-rendering program (Ant, release 2.0.5, Skyscan). 3D measurements were obtained with the CtAn software (release 2.5, Skyscan). Trabecular bone analysis was performed on the femur body. The following 3D parameters were calculated: trabecular volume (BV/TV, in %), trabecuber thickness (Tb.Th, in mm), trabecular separation (Tb.Sp. in μm) and trabecular number (Tb.N, in 1/mm).

### Statistical analysis

Data were shown as mean ± SD for at least three independent experiments. Differences between groups were determined using paired two-sided Student’s *t*-test or two-way ANOVA. Pearson correlation test was used to determine the correlations between gene expressions, and survival analysis and a log-rank test were done by GraphPad Prism 5.0. A *P* value less than 0.05 was considered statistically significant compared with the controls, respectively.

### Reporting summary

Further information on research design is available in the Nature Research Reporting Summary linked to this article.

## Supplementary information

Supplementary Information

Description of Additional Supplementary Files

Supplementary Dataset 1

Supplementary Dataset 2

Supplementary Dataset 3

Reporting Summary

## Data Availability

All data needed to evaluate the conclusions in the paper are present in the paper and/or the Supplementary Materials. All relevant data are available from the authors, and the RNA-seq and ChIP-seq data can be publicly found at the Gene Expression Omnibus database under accession number GSE156872. Source data are provided with this paper. Requests for any materials in this study should be directed to Zhiqiang Liu and obtained through an MTA. Source data are provided with this paper.

## Source data

Source Data
