# Supplementary material for: Targeting NSD2-mediated SRC-3 liquid–liquid phase separation sensitizes bortezomib treatment in multiple myeloma
Source: Nat Commun. 2021 Feb 15;12:1022. doi: 10.1038/s41467-021-21386-y (PMC7884723; doi:10.1038/s41467-021-21386-y)
Supplement: Supplementary file 1 — Supplementary Information [file 41467_2021_21386_MOESM1_ESM.pdf]

Supplementary figure legends

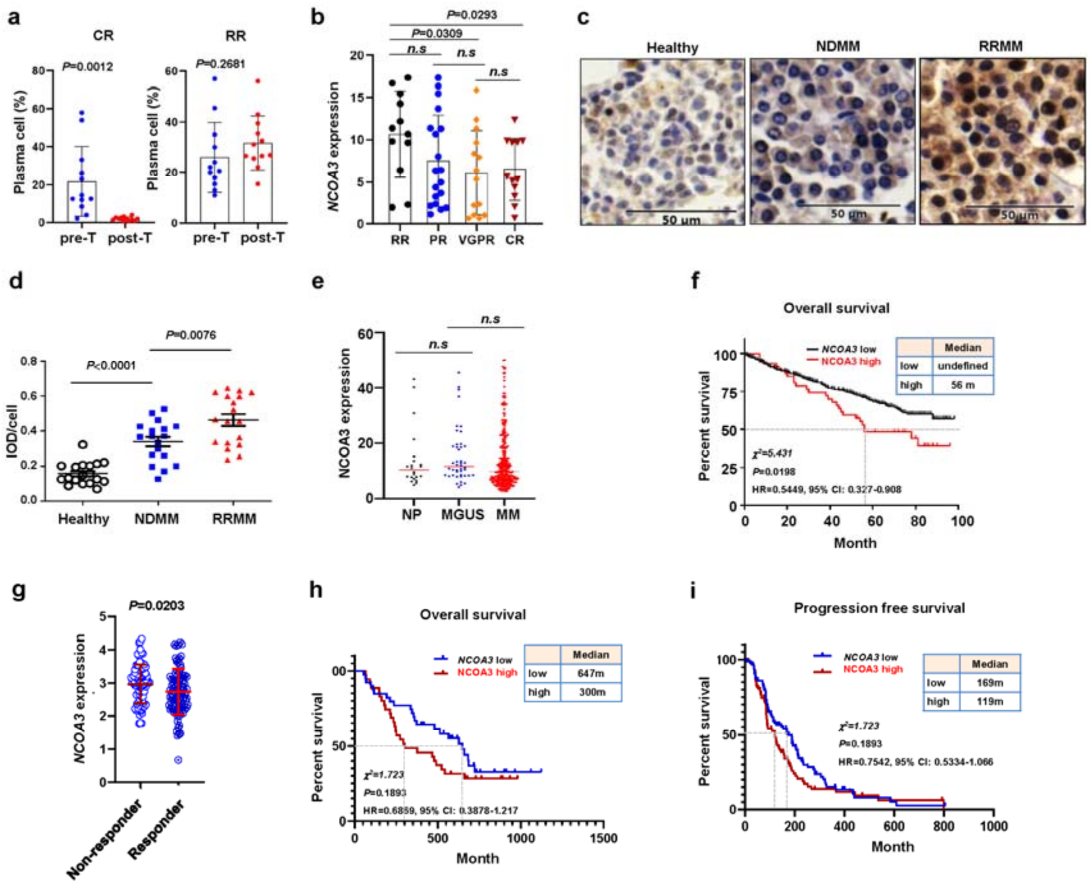

S Figure 1. Significance of SRC-3 expression in multiple myeloma (MM) patients. (**a**) Percentages of bone marrow plasma cell (BMPC) in patients acquired with complete response (CR) (n = 12) and patients of refractory/relapsed (RR) (n = 12). Two-sided *P*-values were determined by Student's *t*-test; mean ± s.d. (**b**) SRC-3 mRNA levels in MM patients with refractory/relapsed (RR, n = 12), partial response (PR, n = 19), very good partial response (VGPR, n = 14), and complete response (RR, n = 12). Two-sided *P*-values were determined by Student's *t*-test; mean ± s.d. (**c**) Representative immunohistochemical staining of SRC-3 protein from healthy controls (n =18), newly diagnosed (NDMM, n = 18) and relapsed myeloma patient (RRMM, n = 18) groups, and (**d**) Integrated Optical Density (IOD) quantification of SRC-3 protein levels using the ImageJ software. Scale bar, 50 µm. Two-sided *P*-values determined by Student's *t* test. mean ± s.d. (**e**) *NCOA3* expressions in plasma cells from healthy controls (n = 22), monoclonal gammopathy of undetermined significance patients (MGUS, n = 44), and the active MM patients (MM, n = 327) in

the cohort of 542 MM patients (GSE2658). Two-sided *P*-values determined by Student's *t* test; n.s., no significance. (**f**) Correlation of SRC-3 expression with overall survival (OS) in the cohort of 542 MM patients after receiving BTZ-based treatment regimens (GSE2658). *P* values by Pearson Coefficient and Log-ranks test. (**g**) SRC-3 expression in MM patients with responses (n = 84) and without response to BTZ-based regimens (n = 85) in GS9782 cohort. Two- sided *P* value by Student's *t*-test; mean ± s.d. (**h**) Correlation of SRC-3 expression with overall survival (OS) and (**i**) progression-free survival (PFS) in 264 patients after receiving BTZ-based treatment regimens. *P* values by Pearson Coefficient and Log-ranks test. Source data are provided as a Source Data file.

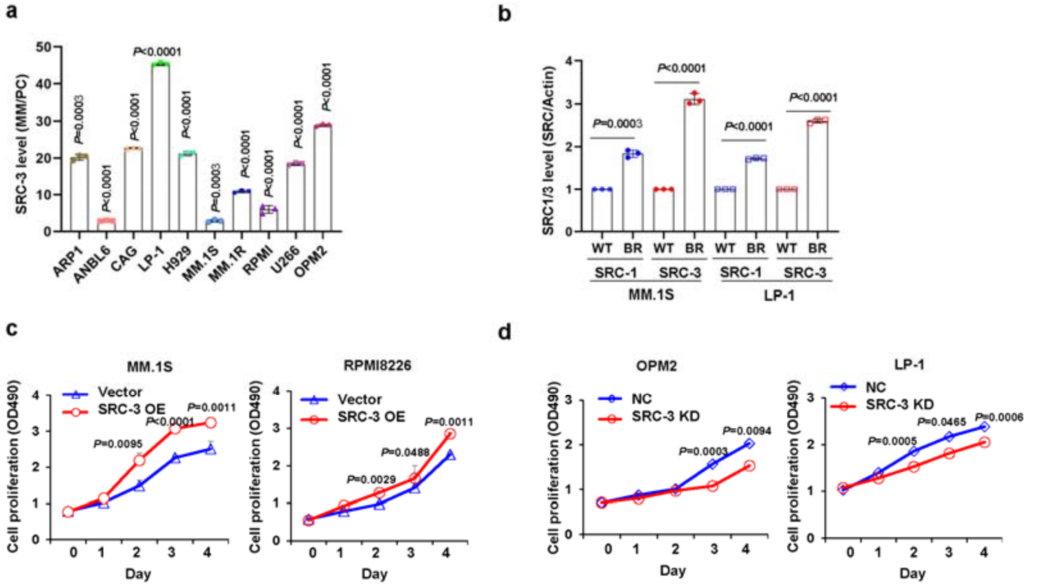

S Figure 2. Levels of SRC-3 in MM cell lines and effects on myeloma cell proliferation. (**a**) Quantification of relative SRC-3 level in ten MM cell lines compared to normal plasma cells (PC) from Fig. 2a (n = 3 biologically independent experiments). Two-sided *P* values by Student's *t* test; mean ± s.d. (**b**) Quantification of SRC-1 and SRC-3 changes in the wild type (WT) or bortezomib (BTZ)-resistant (BR) cells of figure 2g (n = 3 biologically independent experiments). Two-sided *P* values by Student's *t* test; mean ± s.d. (**c**) SRC-3 low expression MM.1S and RPMI8226 cells infected with lentivirus carrying pITA-SRC-3-FLAG (SRC-3 OE) or empty vector (Vector) (n = 3 biologically independent experiments), (**d**) SRC-3 high expression OPM2 and LP-1 cells infected with lentivirus carrying NCOA3-shRNA

(SRC-3 KD) or non-target control (NC) (n = 3 biologically independent experiments) for 3 days were screened by 0.3 µg/mL puromycin, and cell proliferation was determined using MTS kit. Two- sided *P* values by Student's *t* test; mean ± s.d.

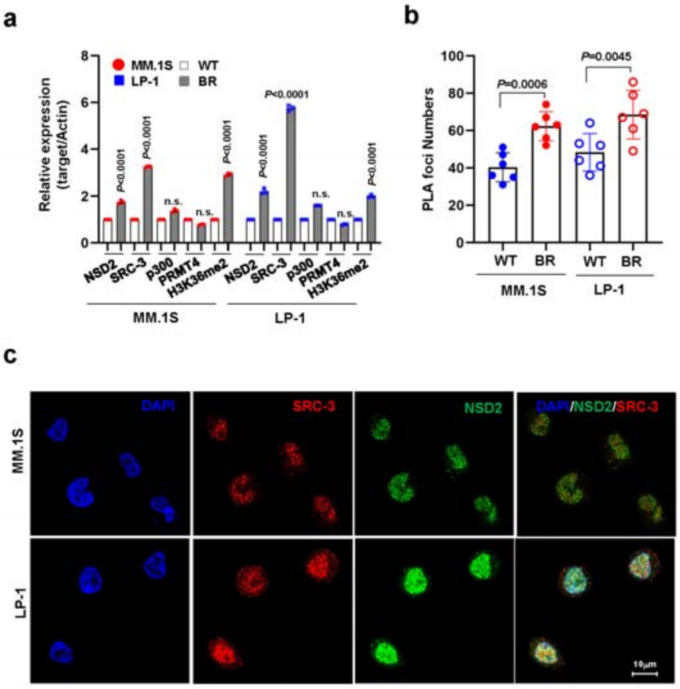

S Figure 3. Co-localization of SRC-3 and NSD2 in myeloma cells.

(**a**) Quantification of changes of SRC-3 and the correlated proteins in the wild type (WT) or bortezomib (BTZ)-resistant (BR) cells from Fig. 4c (n = 3 biologically independent experiments). Two-sided *P* values by Student's *t* test; mean ± s.d. (**b**) Proximity Ligation Assay (PLA) foci numbers were analyzed for SRC-3 in wild type (WT) or bortezomib (BTZ)-resistant (BR) MM.1S and LP-1 cells from Fig. 4d (n = 30 cells from 3 biologically independent experiments). Two-sided *P* values by student *t*-tests, mean ± s.d. (**c**) Immunofluorescence staining (n = 3 biologically independent experiments) to detect the co-localization of endogenous NSD2 and SRC-3 in the MM.1S and LP-1 cells. DAPI (4',6-diamidino-2-phenylindole) was used to identify the nucleus. Scale Bar, 10 µm. Source data are provided as a Source Data file.

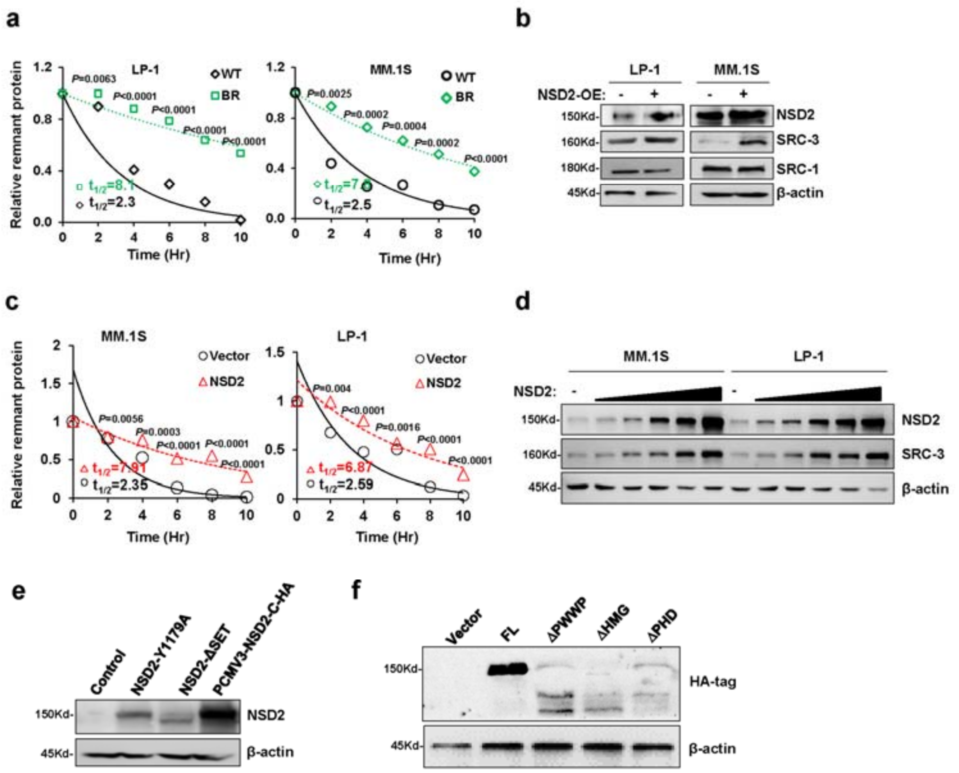

S Figure 4. NSD2 stabilizes SRC-3 in myeloma cells. (**a**) Densitometric analysis for half-life of SRC-3 in the Fig. 4e using Image J software (n = 3 biologically independent experiments). Two-sided *P* values by Student's t test; mean ± s.d. (**b**) Western blotting showing overexpression of NSD2 and the effect on SRC-3 protein level in LP-1 and MM.1S cells (n = 3 biologically independent experiments). (**c**) Densitometric analysis of immunoblots from Fig. 4f for half-life of SRC-3 using Image J software (n = 3 biologically independent experiments). Two-sided *P* values by Student's *t* test; mean ± s.d. (**d**) NSD2 and SRC-3 levels in MM.1S and LP-1 cells infected with lentivirus carrying NSD2 expressing vector for 3 days (n = 3 biologically independent experiments). (**e**) Expressions of full length of NSD2 with Tyrosine to Alanine mutation (Y1197A), truncations with SET domain depletion (ΔSET) and full length of NSD2 (PCMV3-NSD2-C-HA) in HEK293T cells after transfected for 48 hr (n = 3 biologically independent experiments). (**f**) Expressions of full length of NSD2 (FL) and truncations with PWWP domain depletion (ΔPWWP), HMG domain depletion (ΔHMG), PHD domain depletion (ΔPHD) in HEK293T cells after transfected for 48 hr (n = 3 biologically independent experiments). Source data are provided as a Source Data file.

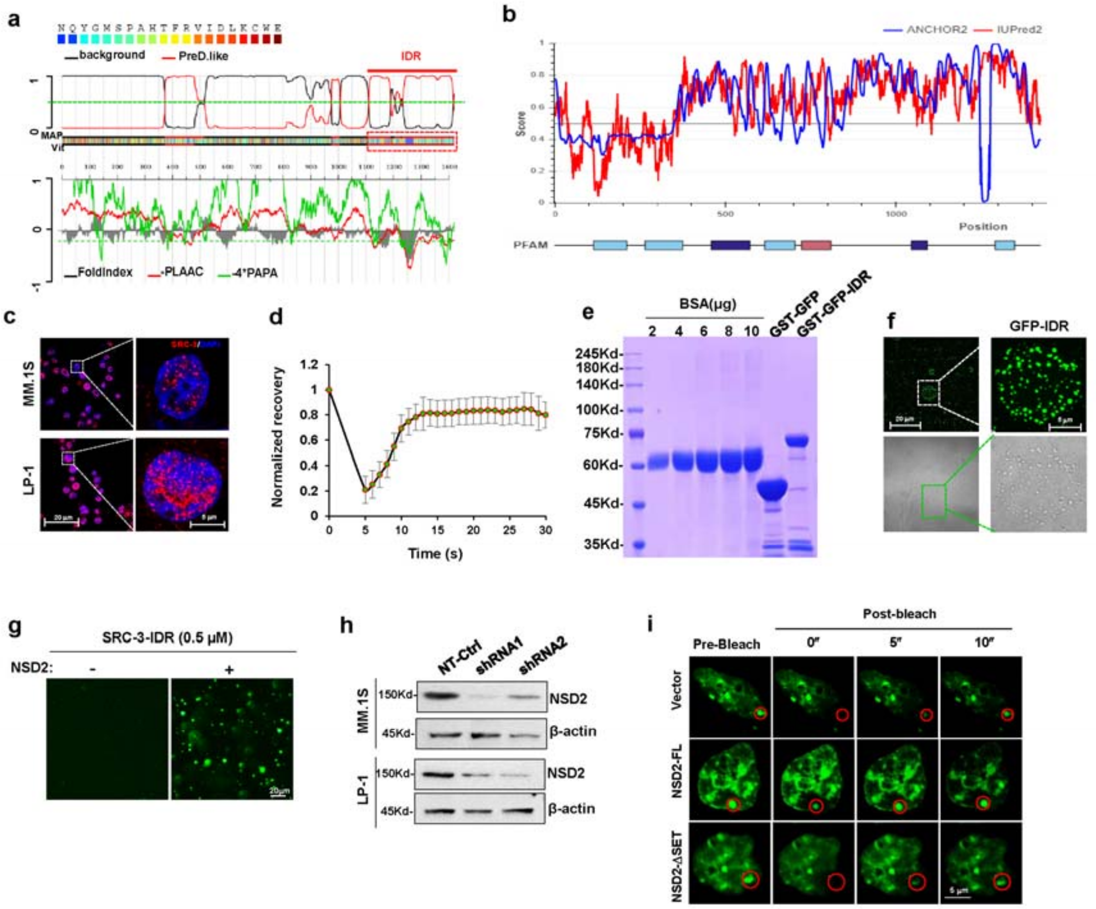

S Figure 5. Prediction of intrinsically disordered regions (IDRs) of SRC-3 using different algorithms. Prediction of the intrinsically disordered regions (IDRs) of SRC-3 protein on (**a**) the PLAAC platform using a hidden-Markov model (HMM) algorithm, (**b**) the IUPred2A web interface that identifies disordered protein regions using IUPred2 and disordered binding regions using ANCHOR2.The red line and the dashed frame indicate the position of IDR on SRC-3 amino acid sequence. The dashed green frames indicated regions are targeted IDRs. (**c**) Representative micrographs (n = 3 biologically independent experiments) of immunofluorescence staining to detect SRC-3 in the MM.1S and LP-1 cells. DAPI (4',6-diamidino-2-phenylindole) was used to identify the nucleus. Scale Bar: left, 20 μm; right, 5 μm. (**d**) Shown are kinetic recovery times of bleached green florescent protein (GFP)-SRC-3 droplet foci in MM.1S. Data are mean ± s.d. of n = 3 biologically independent foci. (**e**) Coomassie blue staining to show in vitro expression of GFP-tagged GST fusion SRC-3 IDR protein. Bovine albumin serum (BSA) was

used as loading control (n = 3 biologically independent experiments). (**f**) *In vitro* liquid phase separation of GFP-IDR fusion protein in presence of 10% PEG 8000 (n = 3 biologically independent experiments). Scale Bar: left, 20 μm; right, 5 μm. (**g**) Representative image of droplet formation of 0.5 μM GFP-SRC-3-IDR in the presence (+) or absence (–) of NSD2 in vitro (n = 3 biologically independent experiments). Scale bar, 20 μm. (**h**) Representative Western blotting (n = 3 biologically independent experiments) showing the knockdown effects in MM.1S and LP-1 cells infected with lentivirus carrying two shRNAs targeting two different coding sequencing of *NSD2* gene (NSD2 KD) compared to the non-target control (NT Ctrl). (**i**) Fluorescence recovery before (pre-bleach) and after photobleaching (FRAP) (post-bleach) of a SRC-3-GFP focus (red circle) by 488 nm laser for 5″ bleaching and 5″, 10″ recovery in MM.1S cells with expressions of vector, full length of NSD2 (NSD2-FL), truncations with SET domain depletion (ΔSET). (n = 3 biologically independent experiments). Scale bar, 5 μm. Source data are provided as a Source Data file.

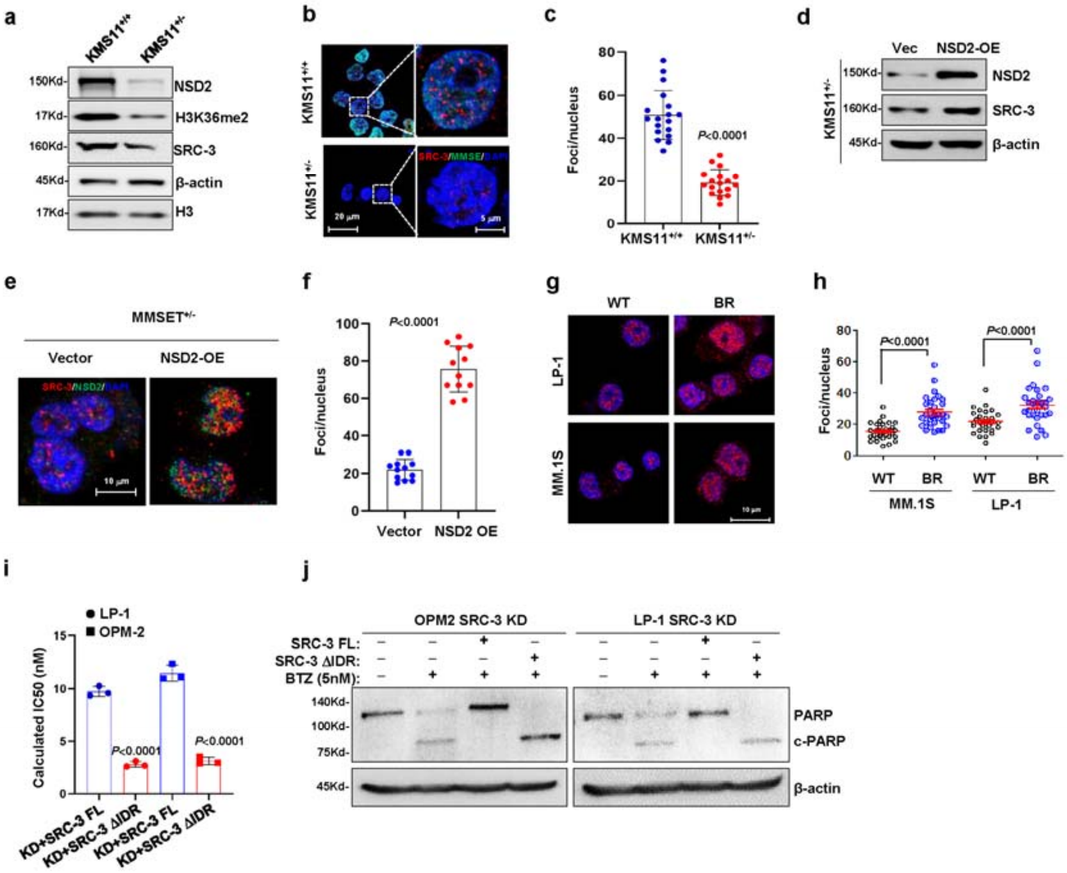

S Figure 6. NSD2 enhances SRC-3 Phase separation.

(**a**) Representative images (n = 3 biologically independent experiments) of Western blotting to show the NSD2, SRC-3, H3K36me2 level in the MMSET$^{+/+}$ and MMSET$^{+/-}$ KMS11 cells. (**b**) Representative images of endogenous SRC-3 foci and NSD2 level in the MMSET$^{+/+}$ and MMSET$^{+/-}$ KMS11 cells determined by immunofluorescence confocal microscopy (n = 3 biologically independent experiments). Scale Bar: left, 20 μm; right, 5 μm. (**c**) Immunofluorescence foci numbers were analyzed for SRC-3 in the MMSET$^{+/+}$ and MMSET$^{+/-}$ KMS11 cells. (n = 12 cells from n =3 biologically independent experiments). Two-sided *P*-value by Student's *t*-test; mean ± s.d. (**d**) Representative Western blotting (n = 3 biologically independent experiments) showing the ectopic expression of NSD2 in MMSET$^{+/-}$ KMS11 cell infected with lentivirus carrying the NSD2-C-HA (NSD2 OE) compared to the vector control (Vec). (**e**) Overexpression of vector control (Vector) and NSD2 (NSD2-OE) in MMSET$^{+/-}$ KMS11 cells by confocal microscopy, and (**f**) analyzed the

foci numbers of SRC-3 (n = 12 biologically independent experiments). Scale bar, 10μm; Two-sided *P* value by Student's *t*-test; mean ± s.d. (**g**) Representative images for the endogenous foci of SRC-3 in wild type (WT) and bortezomib-resistant (BR) MM.1S and LP-1 cells, and (**h**) quantification of SRC-3 foci with each plot (n = 30 from 3 independent experiments). Two-sided *P*-values by Student's *t*-test; mean ± s.d. (**i**) Statistical analysis of IC_{50} to bortezomib (BTZ) in LP-1 SRC-3-KD cells or OPM-2 SRC-3-KD cells, which treated with 25 nM SI-2 or vehicle for 24 hours in the presence of SRC-3 full length (FL) or IDR region depletion truncation (ΔIDR) (n = 3 biologically independent experiments). Two-sided *P*-values by Student's *t*-test; mean ± s.d. (**j**) Representative Western blotting (n = 3 biologically independent experiments) shows the cleavage of PARP in in OPM-2 and LP-1 SRC-3 KD cells overexpressing SRC-3 full length (SRC-3 FL) and IDR region depletion truncation (SRC-3 ΔIDR) treated with 5 nM of bortezomib (BTZ) for 48 hours. Source data are provided as a Source Data file.

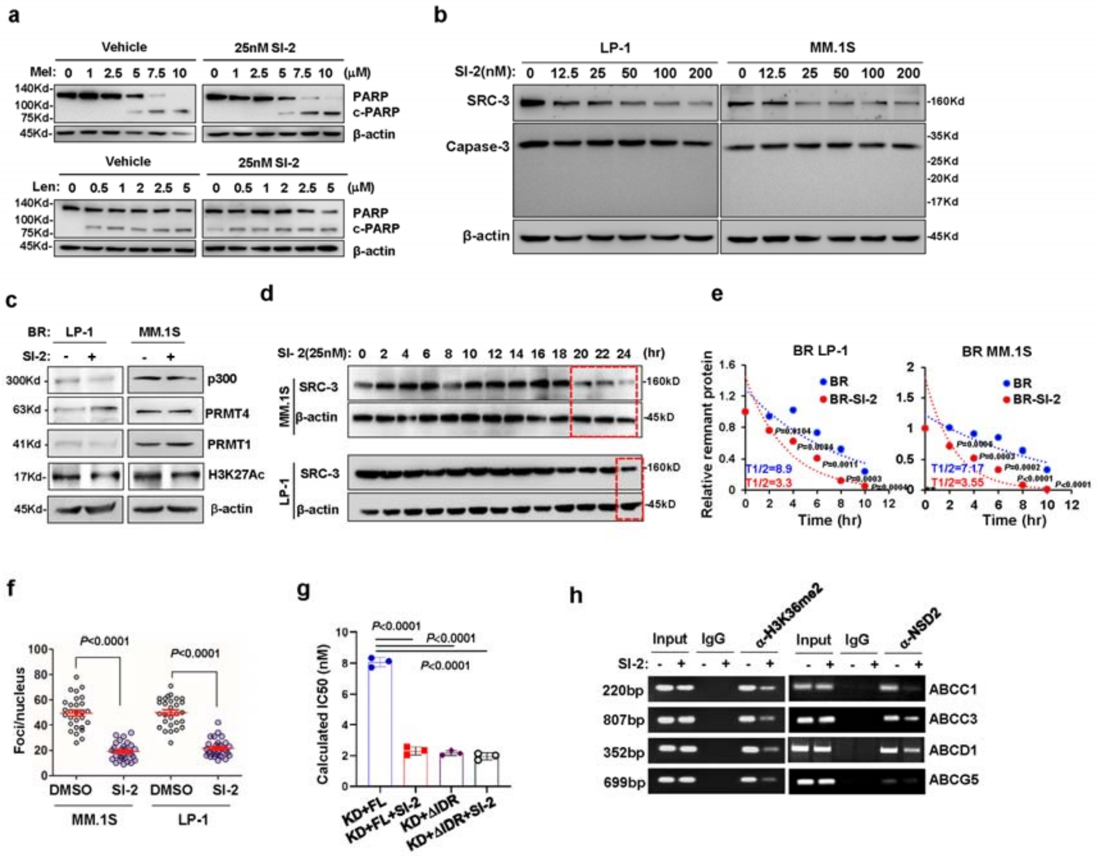

S Figure 7. SI-2 solo administration did not elicit apoptosis in myeloma cells.

(**a**) Representative Western blotting (n = 3 biologically independent experiments) showing the cleavage of PARP in MM cell treated with melphalan (Mel) (0-10 μM) and lenalidomide (Len) (0-5 μM) in the presence or absence of 25 nM SI-2 for 24 hours. (**b**) Representative Western blotting (n = 3 biologically independent experiments) showing cleavage of caspase-3 in MM.1S and LP-1 cells exposed to increasing dosage of SI-2 for 24 hours. (**c**) Representative Western blotting (n = 3 biologically independent experiments) showing p300, PRMT1, PRMT4 and H3K27Ac levels in the bortezomib-resistant (BR) LP-1 and MM.1S cells treated with 25 nM of SI-2 for 24 hours. (**d**) SRC-3 level in MM cell treated with 25 nM SI-2 for 0-24 hours (n = 3 biologically independent experiments). (**e**) Densitometric analysis for the IC_{50} in the MM.1S and LP-1 cells treated with bortezomib (BTZ) alone or BTZ plus SI-2 (n = 3 biologically independent experiments). Two-sided *P* values by Student's *t* test; mean ± SD. (**f**) Endogenous SRC-3 droplet foci in

bortezomib-resistant (BR) cells treated with 25 nM SI-2 or vehicle for 24 hours (n = 30 from 3 independent experiments). Two-sided *P* values by Student's *t*-test; mean ± s.d. (**g**) Statistical analysis of IC_{50} from Fig 6f (n = 3 biologically independent experiments). Two-sided *P* values by Student's t-test; mean ± s.d. (**h**) ChIP-PCR showing enrichment of H3K36me2 and NSD2 on the chromatin of *ABCC1*, *ABCC3*, *ABCD1* and *ABCG5* in bortezomib-resistant (BR) MM.1S cells treated with 25 nM SI-2 for 24 hours (n = 3 biologically independent experiments). Two-sided *P* values by Student's *t*-test; mean ± s.d. Source data are provided as a Source Data file.

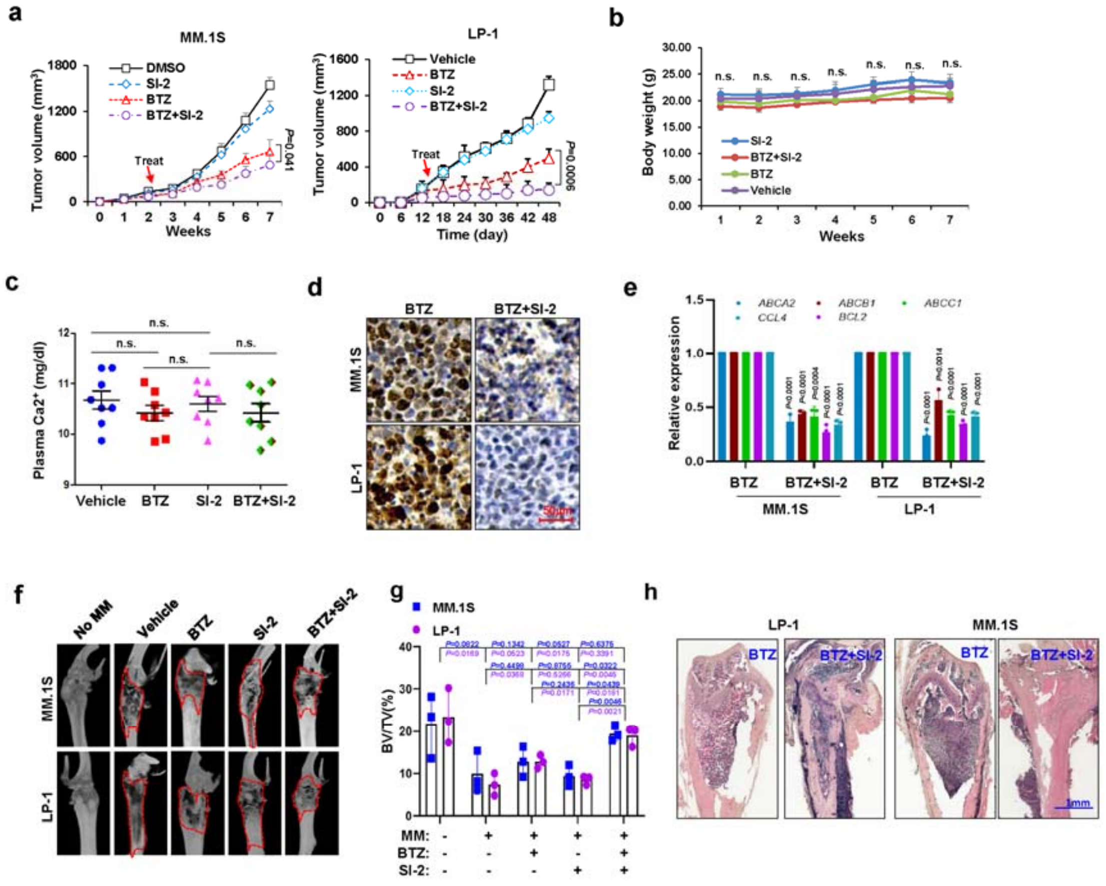

S Figure 8. *In vivo* anti-myeloma effect of bortezomib and SI-2.

(**a**) 2×10^6 MM.1S and LP-1 cells were injected into the left flanking of NSG mice (n = 12 mice per group) and treated with same volume of vehicles, 0.5 mg/kg bortezomib (BTZ), 2 mg/kg SI-2, or BTZ plus SI-2 when tumors were palpable (diameter≈5mm) from week 2 to week 7. Two-sided *P* values by Student's *t*-test was performed; mean ± s.d. (**b**) Body weight and (**c**) plasma calcium level of mice in each

group (n = 8 per group) was monitored very week. Two-sided *P* values by Student *t*-tests; mean ± s.d. (**d**) Immunohistochemistry staining shows the Ki67 level in xenografts tissues from mice (n = 12 per group) treated with bortezomib (BTZ) or BTZ+SI-2. (**e**) qPCR shows the expressions of SRC-3 target genes in xenografts tissues from mice treated with bortezomib (BTZ) or BTZ+SI-2 (n = 3 biologically independent experiments). Two-sided *P* values by student *t*-tests; mean ± s.d. (**f**) Representative microCT reconstructions of mouse femurs bearing MM.1S and LP-1 cells (5×10^5/mouse) and treated with vehicles, 0.5 mg/kg bortezomib (BTZ), 2 mg/kg SI-2, or BTZ plus SI-2 (n = 8 mice) shows osteolytic lesion area, and (**g**) measurement of the percentage of bone volume to total volume (BV/TV); Two-sided *P* values by student *t*-tests; mean ± s.d. (**h**) Haematoxylin Eosin (H&E) staining shows the histological disruption of mice femur bearing bortezomib-resistant (BR) MM.1S and LP-1 cells (5×10^5/mouse) and treated with bortezomib (BTZ) (0.5 mg/kg) or BTZ+SI-2 (n = 8 mice per group). Scale bar, 1 mm. Source data are provided as a Source Data file.

Sequences of all primers

Primers sequence	
HumanGAPDH-F	TTGCCCTCAACGACCACTTT
HumanGAPDH-R	TGGTCCAGGGGTCTTACTCC
HumanNCOA3-F	TCATGAGACCCCGGACAAAC
HumanNCOA3-R	CATTAAGAAAACCCTGCTGGGAG
HumanNCOA1-F	CTGTGTGCCCTGAGCAGATAA
HumanNCOA1-R	CACCTGAACCTGTTGCACCT
HumanABCA2-F	ATCTCCGTGAAGGAAGTCTCC
HumanABCA2-R	GTACTGCAGGAAGCCGAACT
HumanABCA3-F	CTCGCTGTTCCTCAAGCAGA
HumanABCA3-R	AGACCTTCAAACCTGTGCGT
HumanABCB1-F	CTACATGAGAGCGGAGGACAAG
HumanABCB1-R	CTTCCGTTGCACCTCTCTGGT
HumanABCC1-F	GGACTCAGGAGCACACGAAA
HumanABCC1-R	ACGGCGATCCCTTGTGAAAT
HumanBCL2-F	CCTCGCTGCACAAATACTCC
HumanBCL2-R	TGGAGAGAATGTTGGCGTCT
HumanCCL4-F	TGCTGCTTTTCTTACACCGC
HumanCCL4-R	GCAGCTCAGTTCAGTTCCAG
HumanEGR1-F	ACCGCAGAGTCTTTTCCTGA
HumanEGR1-R	CTCACTAGGCCACTGACCAA
HumanIGF1R-F	GAGAGGAGCAGCTAGAAGGG
HumanIGF1R-R	CCCTTTAGTCCCCGTCACTT
HumanFAM53A-F	GGCCCTGATTTCTCCTTCCT
HumanFAM53A-R	CTCCTCGGGTTCTGACAAGG
pITA-hNCOA3-NotI-FLAG-F	TAAGCGGCCGCATGATGGATTACAAGGATGAC GACGATAAGGAGACGTCTGCCTCAGC
pITA-hNCOA3-KpnI-R	TGCTCTAGACTAGGTCATCATGTTCAGGA
pEGFP-C1-SRC3-XhoI-F	CCGCTCGAGAGTGGATTAGGAGAAAACT
pEGFP-C1-SRC3-KpnI-R	CGGGGTACCTCAATCAGAAGAACAGGT
pGEX-6p-1-GFP-SRC3-IDR1-XhoI-F	CCGCTCGAG CTTCAGAGAGAACAGAATGGAT
pGEX-6p-1-GFP-SRC3-IDR1-NotI-R	ATAAGAATGCGGCCGCTCAAATTTCTTCCAGG CCTG
pGEX-6p-1-GFP-SRC3-IDR2-XhoI-F	AATCCGCTCGAGGGACAGGCATTAGAGCCCAA
pGEX-6p-1-GFP-SRC3-IDR2-NotI-R	ATAAGAATGCGGCCGCTCAGCAGTATTTCTGA TCAG
Human NCOA3-RNAi-1	ccTCCGCAACAGTTTCCATAT
Human NCOA3-RNAi-2	gcAGTCTATTCGTCCTCCATA
Human NCOA3-RNAi-3	ccTCTACATCTGGAGGAGTAT

HumanABCC1-F	AGGGCAAAGGCAAAATGCTG
HumanABCC1-R	CAGTTTGGAGGGCTCAGAAG
HumanABCC3-F	GGGGAAGGTGTCCACTAACC
HumanABCC3-R	AGTCAGGTTTGAGTCCCAGC
HumanABCD1-F	CACAGCGTGTGTGAGTGG
HumanABCD1-R	AACATACCACAGGCGTTCCAG
HumanABCG5-F	ACTCCTTGCATTCGCAGTACC
HumanABCG5-R	CACTTCGGGCTCCCTCTTTAG

Key resources of this study

Category	Source	Cat. No.
**Antibodies**		
Anti-rat SRC3(5E11)	Cell signaling technology	2126
Anti-WHSC1/NSD2 antibody [29D1] - ChIP Grade	Abcam	ab75359
Anti-Histone H3 (acetyl K27) antibody- ChIP Grade	Abcam	ab4729
Anti-SRC3 Rabbit	Abcam	ab2831
Anti-Histone H3 (di methyl K36) antibody-ChIP Grade	Abcam	ab9049
Anti-SRC-1 (128E7) Rabbit mAb	Cell signaling technology	2191
Anti-rabbit CBP(D6C6)	Cell signaling technology	7389
Anti-rabbit P300(D8Z4E)	Cell signaling technology	86377
Anti-rabbit PRMT1	Proteintech	11279-1-AP
Anti-mouse PRMT4/CARM1(3H2)	Cell signaling technology	12495
Anti-PARP	Cell signaling technology	9532
Anti-caspase3	Cell signaling technology	9662
Anti-Histone H3 antibody - Nuclear Loading Control and ChIP Grade	Abcam	ab1791
Anti-mouse GAPDH	UTIBODY	UM4002
Anti-rabbit β-actin	Abclonal	AC006
Goat Anti-Rabbit IgG-HRP	Sigma-Aldrich	A0545
ANTI-FLAG® M2-Peroxidase	Sigma-Aldrich	A8592
Rabbit Anti Mouse IgG-HRP	Sigma-Aldrich	A9044-2ML
Anti-rabbit IgG	Proteintech	30000-0-AP
Anti-mouse IgG	Proteintech	B900620
Anti-HA-Tag (C29F4) Rabbit mAb	Cell signaling technology	3724
FITC-conjugated goat anti-rabbit IgG	Abcam	ab97050
**Chemicals, Peptides and Recombinant Proteins**		
3FLAG peptide	Sigma-Aldrich	F4799
FLAG Peptide	Sigma-Aldrich	F3290
Human IL-6 standard	R&D system	840245
1,6-Hexanediol	Sigma-Aldrich	H11807
NaCl	Sigma-Aldrich	S7653
PEG 8000 30% solution	Sigma-Aldrich	86686
**Drugs**		
Bortezomib (PS-341)	SelleckChem	S1013
MG132	SelleckChem	S2619
SI-2	MedKoo	561489
Carfilzomib(PR-171)	SelleckChem	S2853
Puromycin 2HCL	SelleckChem	S7417
Cycloheximide	Sigma-Aldrich	C7698
Melphalan, minimum 95%	Sigma-Aldrich	M2011

Dexamethasone	Sigma-Aldrich	D4902
Lenalidomide	SelleckChem	S1029
Carfilzomib	South San Francisco	CA94080
**Enzymes**		
RNase A, DNase and protease-free	Thermo Fisher	EN0531
Proteinase K Solution, ChIP grade	Thermo Fisher	26160
Benzonase Nuclease	Sigma-Aldrich	E1014-25KU
FastAP Thermosensitive Alkaline Phosphatase	Thermo Fisher	EF0651
BsmBI	NewEngland Biolabs	R0580S
NotI-HF	NewEngland Biolabs	R3189S
BamHI	NewEngland Biolabs	R0136S
XbaI	NewEngland Biolabs	R0145S
EcoRI	NewEngland Biolabs	R0101S
KpnI-HF	NewEngland Biolabs	R3142S
HindIII	NewEngland Biolabs	R104S
XhoI	NewEngland Biolabs	R0146S
CutSmart Buffer	NewEngland Biolabs	137204S
NEBuffer1	NewEngland Biolabs	B7001S
NEBuffer2	NewEngland Biolabs	B7002S
NEBuffer3	NewEngland Biolabs	B7003O
NEBuffer4	NewEngland Biolabs	B7004S
T4 DNA Ligase	NewEngland Biolabs	M0202S
10×Buffer for T4 DNA ligase	NewEngland Biolabs	B0202S
Multiscribe Reverse Transcriptase	ABI	4308228
dNTP mix	ABI	362275
**Plasmids**		
pCMV3-C-HA-NSD2	Sino Biological	HG11530-CY
pCMV3-HA Vector	CLONTECH	S1825
lentiCRISPRv2 puro	Addgene	98290
hU6-MSC-Ubiquitin-EGFP vector	Shanghai genechem	PIEE248068084
NCOA3-shRNA1	Shanghai genechem	PIEE248068084
NCOA3-shRNA2	Shanghai genechem	PIEE248068084
NCOA3-shRNA3	Shanghai genechem	PIEE248068084
pCDH-MSCV-MCS-EF1-copGFP	Gift from Dr. Dr. Robert Orlowski, UT MD Anderson Cancer Center	
pcDNA-3×FALG	Gift from Dr. Michael Naksi lab, UT Health Science Center at San Antonio	
pITA insert	Gift from Dr. Yupeng Chen, Tianjin Medical University	
$PSPAX_2$	Gift from Dr. Xudong Wu, Tianjin Medical University, Dept. Cell Biology	
PMD_2G	Gift from Dr. Xudong Wu, Tianjin Medical University, Dept. Cell Biology	

pEGFP-C1	Gift from Dr. Yupeng Chen, Tianjin Medical University	
pGEX-6p-1-GFP	Gift from Dr. Yupeng Chen, Tianjin Medical University	
pITA insert-SRC3-FLAG	Self-construction	
pITA insert-SRC3-ΔIDR-FLAG	Self-construction	
pEGFP-C1-SRC3	Self-construction	
pGEX-6p-1-GFP-SRC3-IDR1	Self-construction	
pGEX-6p-1-GFP-SRC3-IDR2	Self-construction	
pCMV3-C-HA-NSD2ΔPWWP	Self-construction	
pCMV3-C-HA-NSD2ΔHMG-box	Self-construction	
pCMV3-C-HA-NSD2ΔPHD	Self-construction	
pCMV3-C-HA-NSD2ΔSET	Self-construction	
pCMV3-C-HA-NSD2-Y1179A	Self-construction	
MMSET sgRNA1(lentiCRISPR)	Self-construction	
MMSET sgRNA2(lentiCRISPR)	Self-construction	
MMSET sgRNA3(lentiCRISPR)	Self-construction	
**Critical Commercial Assays**		
EvaGreen 2X qPCR MasterMix	ABI	MasterMix-R
5×All-In-One RT MasterMix	abm	G490
Luciferase assay Kit	Promega	E1910
Duolink™ In Situ Detection Reagents Red	Sigma-Aldrich	DUO92008
Pierce BCA Protein Assay Kit	Thermo SCIENTIFIC	23225
AxyPrep DNA Extraction Kit	AXYGEN	295 AP-GX-250G
AxyPrep Plasmid Miniprep Kit	AXYGEN	183 AP-MN-P-250G
Plasmid Maxi Kit(25)	QIAGEN	12163
EnVision G12 Doublestain System,Rabbit/Mouse(DAB+/Permanent Red)	Dako	K5361
SuperSignal West Dura Extended Duration Substrate	ThermoFisher	34580
9002 SimpleCHIP® Kit	Cell Signaling	22188S
Simple CHIP® Kits-20C-Reagents	Cell Signaling	45061S
ChIP-grade Protein A/G Magnetic Beads	Thermo SCIENTIFIC	26162
Lipofectamine 3000 Transfection Kit	Invitrogen	L3000-008
ANTI-FLAG M2 Affinity Gel	Sigma-Aldrich	A2220
CellTiter 96 Aqueous One Solution	Promega	G358B
NuPAGE 4-12% Bis-Tris Gel	Invitrogen	NP0335BOX
Phosphatase Inhibitor Cocktail(100×)	Cell Signaling	587OS
Annexin V-FITC Apoptosis Kit	Sigma-Aldrich	APOAF-50TST
Pierce® Protein G Plus Agarose	Thermo Scientific	22852
Human CD20 MicroBeads	Miltenyi Biotec	MB17-R0829
Human CD138 MicroBeads	Miltenyi Biotec	130-105-961

Whole Blood Column Kit	Miltenyi Biotec	MB17-R0189
DeadEnd™ Fluorometric TUNEL System	Promaga	G3250
Tartrate-resistant acid phosphatase (TRAP) Kit	Solarbio	G1492
Ficoll-Paque PLUS endotoxin tested	GE Healthcare	17-1440-02
LS Columns(25 columns)	Miltenyi Biotec	130-042-401
TRIzol Reagent	Ambion, Life Science	15596018
Opti-MEM®I(1×) Reduced Serum	Gibco, Life Technologies	31985-070
Opti-protein XL Marker	ABM	G266
PageRuler Prestained protein Ladder	ThermoFisher Scientific	26616
1Kb Ladder DNA Marker	Biomed	MD114
1Kb DNA Ladder	TIANCEN	MD111
100bp DNA Ladder	TRANS	BM301
BM15000 DNA Marker	Biomed	MD106
1Kb Plus DNA Ladder	Solarbio	M1500
PEI-Transferrinfection Kit	ThermoFisher Scientific	BMS1003
