# Supplementary material for: Targeting NSD2-mediated SRC-3 liquid–liquid phase separation sensitizes bortezomib treatment in multiple myeloma
Source: Nat Commun. 2021 Feb 15;12:1022. doi: 10.1038/s41467-021-21386-y (PMC7884723; doi:10.1038/s41467-021-21386-y)
Supplement: Supplementary file 2 — Description of Additional Supplementary Files [file 41467_2021_21386_MOESM2_ESM.pdf]

Description of Additional Supplementary Files

Title: Supplementary Data 1.

Description: SILAC screening of differentially expressed proteins

Title: Supplementary Data 2.

Description: Differentially expressed genes in WT and BR myeloma cells.

Title: Supplementary Data 3.

Description: Differentially expressed genes in SI-2 treated BR myeloma cells.
